# Prognostic value of integrated cytogenetic, somatic variation, and copy number variation analyses in Korean patients with newly diagnosed multiple myeloma

**DOI:** 10.1371/journal.pone.0246322

**Published:** 2021-02-05

**Authors:** Nuri Lee, Sung-Min Kim, Youngeun Lee, Dajeong Jeong, Jiwon Yun, Sohee Ryu, Sung-Soo Yoon, Yong-Oon Ahn, Sang Mee Hwang, Dong Soon Lee

**Affiliations:** 1 Department of Laboratory Medicine, Kangnam Sacred Heart Hospital, Hallym University College of Medicine, Seoul, Korea; 2 Cancer Research Institute, Seoul National University College of Medicine, Seoul, Korea; 3 Department of Laboratory Medicine, Seoul National University Hospital, Seoul, Korea; 4 Department of Internal Medicine, Clinical Research Institute, Seoul National University Hospital, Cancer Research Institute, Seoul National University, College of Medicine, Seoul, Korea; 5 Department of Laboratory Medicine, Seoul National University Bundang Hospital, Seongnam, Korea; UCSI University, MALAYSIA

## Abstract

**Background:**

To investigate the prognostic value of gene variants and copy number variations (CNVs) in patients with newly diagnosed multiple myeloma (NDMM), an integrative genomic analysis was performed.

**Methods:**

Sixty-seven patients with NDMM exhibiting more than 60% plasma cells in the bone marrow aspirate were enrolled in the study. Whole-exome sequencing was conducted on bone marrow nucleated cells. Mutation and CNV analyses were performed using the CNVkit and Nexus Copy Number software. In addition, karyotype and fluorescent in situ hybridization were utilized for the integrated analysis.

**Results:**

Eighty-three driver gene mutations were detected in 63 patients with NDMM. The median number of mutations per patient was 2.0 (95% confidence interval [CI] = 2.0–3.0, range = 0–8). *MAML2* and *BHLHE41* mutations were associated with decreased survival. CNVs were detected in 56 patients (72.7%; 56/67). The median number of CNVs per patient was 6.0 (95% CI = 5.7–7.0; range = 0–16). Among the CNVs, 1q gain, 6p gain, 6q loss, 8p loss, and 13q loss were associated with decreased survival. Additionally, 1q gain and 6p gain were independent adverse prognostic factors. Increased numbers of CNVs and driver gene mutations were associated with poor clinical outcomes. Cluster analysis revealed that patients with the highest number of driver mutations along with 1q gain, 6p gain, and 13q loss exhibited the poorest prognosis.

**Conclusions:**

In addition to the known prognostic factors, the integrated analysis of genetic variations and CNVs could contribute to prognostic stratification of patients with NDMM.

## Introduction

Multiple myeloma (MM) is the second most common hematological malignancy in Korea and the USA [1–3]. The treatment outcomes of patients with MM have markedly improved owing to the development of novel therapeutic agents and the technical advances in molecular diagnostics and detection of minimal residual diseases [4, 5]. There is a consensus on the risk factors that aid in stratifying patients with MM. The methods to detect genetic variations include conventional karyotyping and fluorescent in situ hybridization (FISH) [6, 7].

Recently, the genetic landscape of MM has expanded owing to the use of high-throughput technologies, such as next-generation sequencing for analyzing targeted genes and whole-exome or genome [8–11]. Previous studies have revealed novel and frequently mutated genes, such as *KRAS*, *NRAS*, *BRAF*, and *FAM46C* in MM. Additionally, the chronological evolution of multiple driver events has been demonstrated using serial patient specimens [8]. However, the prognostic effect of most mutated genes, which have a low recurrence rate, has not been clearly identified [12]. There are limited studies on the comprehensive analysis of various cancer driver events and the correlation between structural variants and genomic events.

Recent studies have performed clustering analysis that integrates various prognosis-related genomic variations to classify patients with MM based on the characteristics of genomic alterations [8–10]. The heterogeneous nature of MM has warranted further studies on the multifaceted interpretation of subgroups incorporating various genetic variations and their prognostic relevance. Several studies have utilized emerging technologies to examine the prognostic implications of genetic variations. However, there are no consensus guidelines that include somatic variations and small structural variations.

In this study, the genomic profile of Korean patients with newly diagnosed MM (NDMM) was examined using whole-exome sequencing (WES) and new prognosis subgroups were identified through integrated analysis of copy number variations (CNVs) and somatic variants.

## Materials and methods

### Patients

In this study, 67 patients with NDMM exhibiting more than 60% plasma cells in the bone marrow (BM) aspiration were recruited at the Seoul National University Hospital between July 2004 and January 2017. Patients were diagnosed to NDMM based on BM aspiration and biopsy according to the international myeloma working group (IMWG) 2016 guideline [13]. A total of three laboratory hematologists reviewed the slide for diagnosis and differential counting of plasma cells. Patients with >60% plasma cells were included in this study to maximize representativeness of variants for genetic changes of plasma cells. In particular, since the revised IWMG guideline designated plasma cell count over 60% as biomarkers of active myeloma, 60% was established as the selection criteria. The clinical characteristics, including the age of disease onset, sex, CRAB (hypercalcemia, renal impairment, anemia, and bone disease) symptoms, chemotherapy regimens, and survival, as well as the laboratory findings, including complete blood count, blood urea nitrogen/creatinine, albumin, lactate dehydrogenase levels, BM histological findings, and cytogenetic findings (FISH and conventional cytogenetics [CG]), of each patient were recorded. This study was approved by the Institutional Review Board of Seoul National University Hospital (IRB No. 1312-102-544). All study subjects provided their written informed consent to participate in the study.

### DNA extraction and exome sequencing

DNA isolated from the frozen BM mononuclear cells was used in the exome capture protocol. The SureSelectHuman All Exon V5+UTR probe set included 359,555 exons of 21,522 genes, and the size of the total targeted region was 75 Mb. To generate the standard exome capture libraries, the Agilent SureSelect Target Enrichment protocol was used for generating the Illumina paired-end sequencing library (ver. B.3, June 2015) with 3 μg input genomic DNA (gDNA). The quantity and quality of DNA were examined using PicoGreen (Molecular Probes, Eugene, OR) and Nanodrop (ThermoFisher Scientific, Mississauga, ON). DNA concentration ≥ 50 ng/uL, purity ≥ 1.7 (A260/A280), volumn ≥ 20 uL and total amount ≥ 1 ug was passed for quality control criteria, and determined to acceptable for evaluation. The gDNA (1 μg) was fragmented using adaptive focused acoustic technology (AFA; Covaris). The fragmented DNA was repaired with an adenine ligated to the 3′ end, followed by ligation of the Agilent adapters. The adapter-ligated product was subjected to polymerase chain reaction (PCR). The final purified product was then quantified using quantitative real-time PCR (qRT-PCR) following the qPCR Quantification Protocol Guide and subjected to quality control using the Caliper LabChipHigh Sensitivity DNA (PerkinElmer). For exome capture, 250 ng of all exon capture libraries were mixed with hybridization buffers, blocking mixes, RNase block, and 5 μL of SureSelect, following the standard Agilent SureSelect Target Enrichment protocol. Hybridization to the capture baits was performed at 65°C using a heated thermal cycler with the lid temperature maintained at 105°C for 24 h in a PCR machine. The captured DNA was amplified and the final purified product was quantified using qRT-PCR according to the qPCR Quantification Protocol Guide. The amplified product was subjected to quality control using the TapeStationDNAscreentape (Agilent). The pooled DNA libraries were sequenced using the NovaSeq6000platform (Illumina, San Diego, USA), conducted by outsourced service from Macrogen Inc. (Seoul, South Korea).

### Bioinformatic evaluation of sequencing data

FASTQ files were aligned to the reference human genome (hg19; GRC37) using the Burrows-Wheeler aligner (BWA, v0.62) [14]. Duplicate PCR reads were removed using Picard 1.98. Variant calling was performed using the “HaplotypeCaller” in Genome Analysis Toolkit 2.7–2 [15]. To detect the candidate gene mutations, a filtering strategy was used. Low-quality variants with a low total depth (<20) and a low altered allele count (<10) were filtered out. Synonymous and noncoding variants were discarded. The variants with an allele frequency of more than 0.01 when compared with those in the 1000 Genomes Project (http://browser.1000genomes.org/), the Exome Aggregation Consortium (http://exac.broadinstitute.org/), and the NHLBI exome sequencing project (ESP6500, http://evs.gs.washington.edu/EVS/) databases were excluded. As a matched control sample was not included in this study, a stringent variant selection pipeline was applied to prioritize the high-confidence set of somatic mutations. The driver genes were selected based on two previous studies [8, 10]. Genes that were identified as driver genes in at least one of these two studies were selected as driver genes for this study (S1 Table in S1 File). Additionally, the in silico prediction algorithms, SIFT [16, 17], CADD [18] and PolyPhen2 [19] were used, and the clinical significance was interpreted according to the ACMG guidelines [20].

### Copy number analysis

Copy number alterations were analyzed using a CNVkit [21] and Nexus software version 5 (Biodiscovery, El Segundo, CA). The copy number in the NDMM dataset was called against an MM-negative karyotype FISH panel. Heatmap plots were drawn with the “heatmap” command in CNVkit. Data were loaded into Nexus 5.0 and the copy number calls were generated genome-wide for each sample based on the fixed thresholds for deletions and duplications specified in the settings. SNP‐Rank Segmentation Algorithm [22], a statistics-based algorithm similar to the circular binary segmentation, applies both the copy number value and the B‐allele frequency to the segmentation (S1 Fig in S1 File). GISTIC algorithms were used to determine the significance of focal somatic copy number alterations.

### Statistical analysis

All statistical analyses were performed using the statistical R-project program (version 3.6.2), PASW statistics version 18 (SPSS Inc., Chicago, USA), and MedCalc version 12.0 (MedCalc Software, Mariakerke, Belgium). A pairwise correlation analysis between somatic mutations and CNVs was performed using the Fisher test. Cumulative overall survival (OS) curves for the groups with or without genomic variations were calculated using the Kaplan-Meier (KM) method and compared using the log-rank test and Breslow test. The Cox proportional hazards model was used to evaluate the prognostic impact of CNVs and mutated genes on OS. A multivariate analysis was performed on the full set of significant variables in the univariate analysis. The differences were considered significant at *P* < 0.05.

## Results

### Characteristics of the study population

The demographic and clinical characteristics of the study population, including the laboratory tests performed on the day of MM diagnosis, are described in Table 1. The numbers of male and female patients were 34 and 33, respectively. The median age of the study population was 65 years. Among the total nucleated cells in the BM aspiration, the median percentage of BM plasma cells was 75.8%. The numbers of patients with ISS stage I, II, and III tumors were 7, 25, and 33, respectively (Table 1).

**Table 1:** Demographic and clinical characteristics of the study population.

Variables	Baseline distribution of patients (N = 67)
Sex (Male/Female)	34 (50.7%) / 33 (49.3%)
Age (year)	65.0 (58.0–71.0)
Stage ISS (I/II/III/NA)	7 (10.4%) / 25 (37.3%) / 33 (49.3%) / 2 (3.0%)
Serum.M protein (g/dL)	6.1 (4.3–9.1)
β2-Microglobulin (mg/dL)	6.4 (3.7–15.3)
Hemoglobin (g/dL)	8.3 (7.3–9.3)
Calcium (mg/dL)	9.4 (8.6–10.0)
BUN (mg/dL)	17.0 (14.0–24.8)
Creatinine (mg/dL)	1.1 (0.9–1.9)
Albumin (mg/dL)	3.1 (2.7–3.5)
LDH (U/L)	174 (137.5–221)
Bone.disease	45 (67.1%)
BM plasma.cell (%)	75.8 (66.2–85.4)
Death (Yes/No)	31 (46.3%) / 36 (53.7%)
Treatment	
VAD	14 (21.9%)
VMP	13 (20.3%)
VTD	7 (10.9%)
TD	11 (17.2%)
MP(MPT)	8 (12.5%)
Others*	14 (20.9%)

Values are presented as median (Interquartile Range).

Abbreviations: NDMM, newly diagnosed multiple myeloma; ISS, International staging system; NA, Not available; LDH, Lactate dehydrogenase; BM, Bone marrow; VAD, vincristine/doxorubicin/dexamethasone; VMP, bortezomib/ melphalan/prednisolone; VTD, bortezomib/ thalidomide/dexamethasone; TD, thalidomide/dexamethasone; MPT, melphalan/prednisolone/thalidomide.

*Other: four patients for bortezom.

ib/dexamethasone, four patients for dexamethasone, two patients for ixzomib, one patients for lenalidomide, three patients are unknown for treatment regimen.

### Identification of CNVs in MM

**Fig 1:**
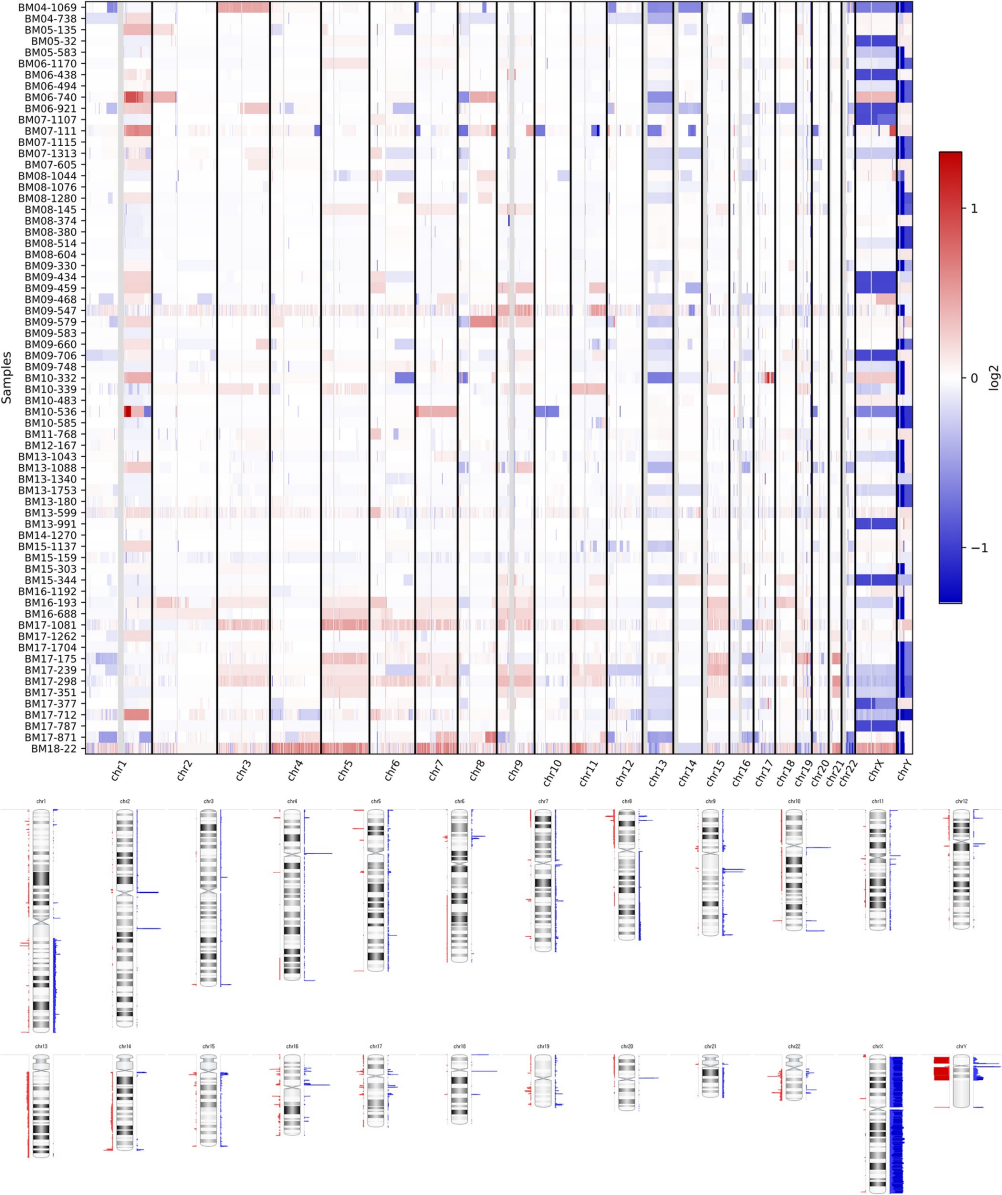
Summary plot of chromosomal gain and loss in 67 patients with newly diagnosed multiple myeloma determined using whole-exome sequencing and copy number analysis. (A) In the heatmap plot, each row represents one patient, while each column represents chromosomes 1–23, X, and Y in order. (B) Ideogram with regions of chromosomal gain and loss. Copy number gains and deletions are marked as blue and red bars to the right and left side of the ideogram, respectively.

The gain and loss of the p arm, q arm, and both arms of chromosomes 1 to 22 were analyzed. The most common chromosomal gain was 1q. Among the odd-numbered chromosomes, the most frequent whole-arm gain was observed in chromosomes 5, 7, 9, 15, and 19. The most common chromosome loss was 13q loss, followed by the losses of 16q, 22q, 8p, 14q, 1p, and 6q (Table 2; Fig 1). The frequency of the gain and loss of each chromosome is represented in S2 Table in S1 File.

**Table 2:** Frequency of copy number alterations in patients with newly diagnosed multiple myeloma.

Chr	Gain				Loss			
	p arm	q arm	whole arms	Total (%)	p arm	q arm	whole arms	Total (%)
1	0	35	0	35 (17.5)	13	3	0	16 (8.1)
2	3	2	1	6 (3.0)	1	5	0	6 (3.0)
3	0	4	8	12 (6.0)	0	0	0	0 (0.0)
4	1	1	1	3 (1.5)	5	4	0	9 (4.5)
5	1	0	14	15 (7.5)	0	1	0	1 (0.5)
6	10	2	4	16 (8.0)	2	12	0	14 (7.1)
7	0	4	11	15 (7.5)	4	0	0	4 (2.0)
8	0	8	0	8 (4.0)	15	1	1	17 (8.1)
9	1	3	14	18 (9.5)	2	0	0	2 (1.0)
10	0	0	0	0 (0.0)	2	2	0	4 (2.0)
11	1	6	6	13 (6.5)	2	4	1	7 (3.5)
12	1	0	0	1 (0.5)	8	1	1	10 (5.1)
13	0	0	0	0 (0.0)	0	33	0	33 (16.7)
14	0	2	0	2 (1.0)	0	13	0	13 (6.6)
15	0	2	12	14 (7.0)	0	0	0	0 (0.0)
16	2	1	0	3 (1.5)	2	18	0	20 (10.1)
17	0	2	1	3 (1.5)	3	2	0	5 (2.5)
18	0	3	6	9 (4.5)	1	0	1	2 (1.0)
19	6	0	11	17 (8.5)	5	0	4	9 (4.5)
20	0	0	0	0 (0.0)	7	3	0	10 (5.1)
21	0	1	6	7 (3.5)	0	0	0	0 (0.0)
22	0	2	0	2 (1.0)	1	16	0	17 (8.6)
Total	26	78	95	199 (100)	73	118	8	199 (100)

### Analysis of selected driver gene mutations

**Fig 2:**
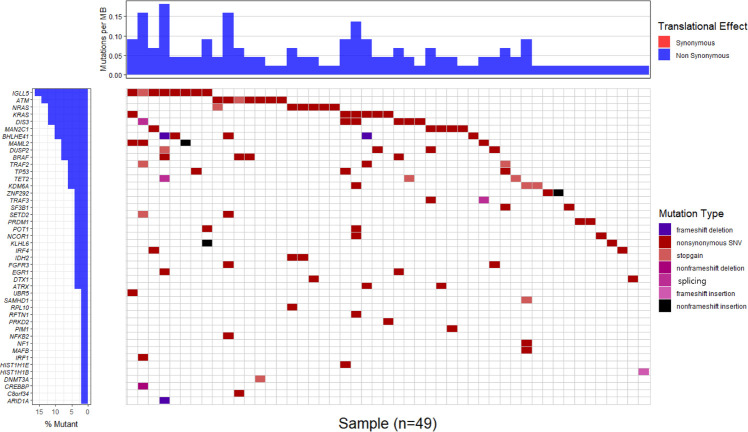
Frequency and distribution of somatic mutations in patients with NDMM. Mutation burden per megabase (Mb) in tumor are represented (upper) and mutated genes are ranked by mutant frequency (lower). The heatmap showed individual mutations in patient samples, color-coded by type of mutation. Genes with significant somatic mutations (pathogenic, likely pathogenic, and variants of unknown significance by ACMG guideline) are represented.

Driver gene mutations were detected in 59 of the 83 genes selected based on two previous studies [8, 10]. Variations classified as pathogenic, likely pathogenic, and variants of unknown significance (VUS) were separately selected based on the ACMG guideline to analyze the frequency and mutation types of each gene (Fig 2). The most frequently mutated driver gene was *IGLL5*, which was detected in eight patients. Additionally, seven patients had mutations in *ATM*, six patients had mutations in *NRAS*, *KRAS*, and *DIS3*, five patients had mutations in *MAN2C1* and *BHLHE41*, four patients had mutations in *MAML2*, *DUSP2*, and *BRAF*, and three patients had mutations in *TRAF2*, *TP53*, *TET2*, and *KDM6A*. The diagram including the variation and domain for each gene is shown in S2 Fig in S1 File. The p.V600E variation in *BRAF* was detected in three patients, while the p.Q61R(L) variation in *KRAS* was detected in three patients. In *NRAS*, the p.G12D(V) and p.Q61L(K) variations were detected in two patients. Mutations, such as p.S403L, p.L581V, and p.Q584X, were detected in *ATM* (S2 Fig in S1 File).

### Correlation analysis of genomic variants

**Fig 3:**
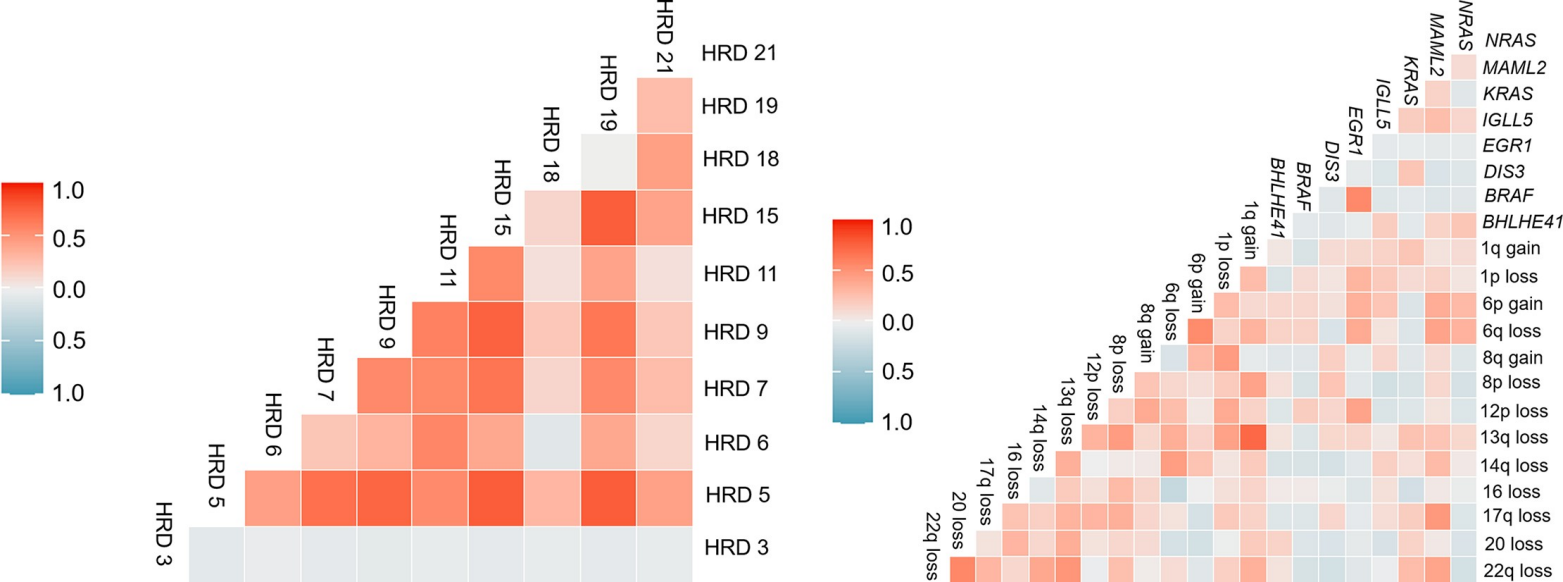
Pairwise associations between mutations and copy number variations. (A) In patients with hyperdiploid multiple myeloma, chromosome 5 variations were correlated with variations in chromosomes 9, 15, and 19, while chromosome 15 variations were correlated with variations in chromosomes 9 and 19. (B) Among patients without hyperdiploid multiple myeloma, 1q gain was correlated with 13q loss, *BRAF* mutation was correlated with *EGR1* mutation, and *MAML2* mutation was correlated with 17q loss.

CNVs were frequently detected with moderate or high correlation between each other. In patients with hyperdiploid MM, CNVs in odd-numbered chromosomes were highly correlated with each other. The CNVs between the following chromosome pairs that occurred simultaneously were highly correlated: chromosomes 5 and 9 (Pearson correlation coefficient (PCC) = 0.745; *P* < 0.001), chromosomes 5 and 15 (PCC = 0.791; *P* < 0.001), chromosomes 5 and 19 (PCC = 0.791; *P* < 0.001), chromosomes 9 and 15 (PCC = 0.763; *P* < 0.001), and chromosomes 15 and 19 (PCC = 0.782; *P* < 0.001) (Fig 3A). Among patients with non-hyperdiploid MM, there was a high correlation between 1q gain and 13q loss (PCC = 0.734, *P* <0.001), and a moderate correlation between the following CNV pairs: 20 loss and 22q loss (PCC = 0.577, *P* <0.001); 6p gain and 6q loss (PCC = 0.553 and *P* <0.001). The analysis of correlations between genes with significant prognostic value among driver gene mutations revealed a significant correlation between *BRAF* and *EGR1* mutations (PCC = 0.564, P <0.001). Additionally, the analysis of the correlation between CNVs and driver gene mutations revealed a significant correlation of *MAML2* mutation with 17q loss (PCC = 0.485, *P* <0.001), 22q loss (PCC = 0.419, *P* = 0.004), 6q loss (PCC = 0.423, *P* = 0.004), and 6p gain (PCC = 0.373, *P* = 0.001). Additionally, *EGR1* mutation was significantly correlated with 12p loss (PCC = 0.426, *P* = 0.004) and 1p loss (PCC = 0.324, *P* = 0.03). The other driver gene mutations were not significantly correlated with CNVs (Fig 3B).

### Prognostic impact of CNVs and somatic mutations in patients with MM

#### Prognostic impact of each of the CNVs and somatic mutations

**Fig 4:**
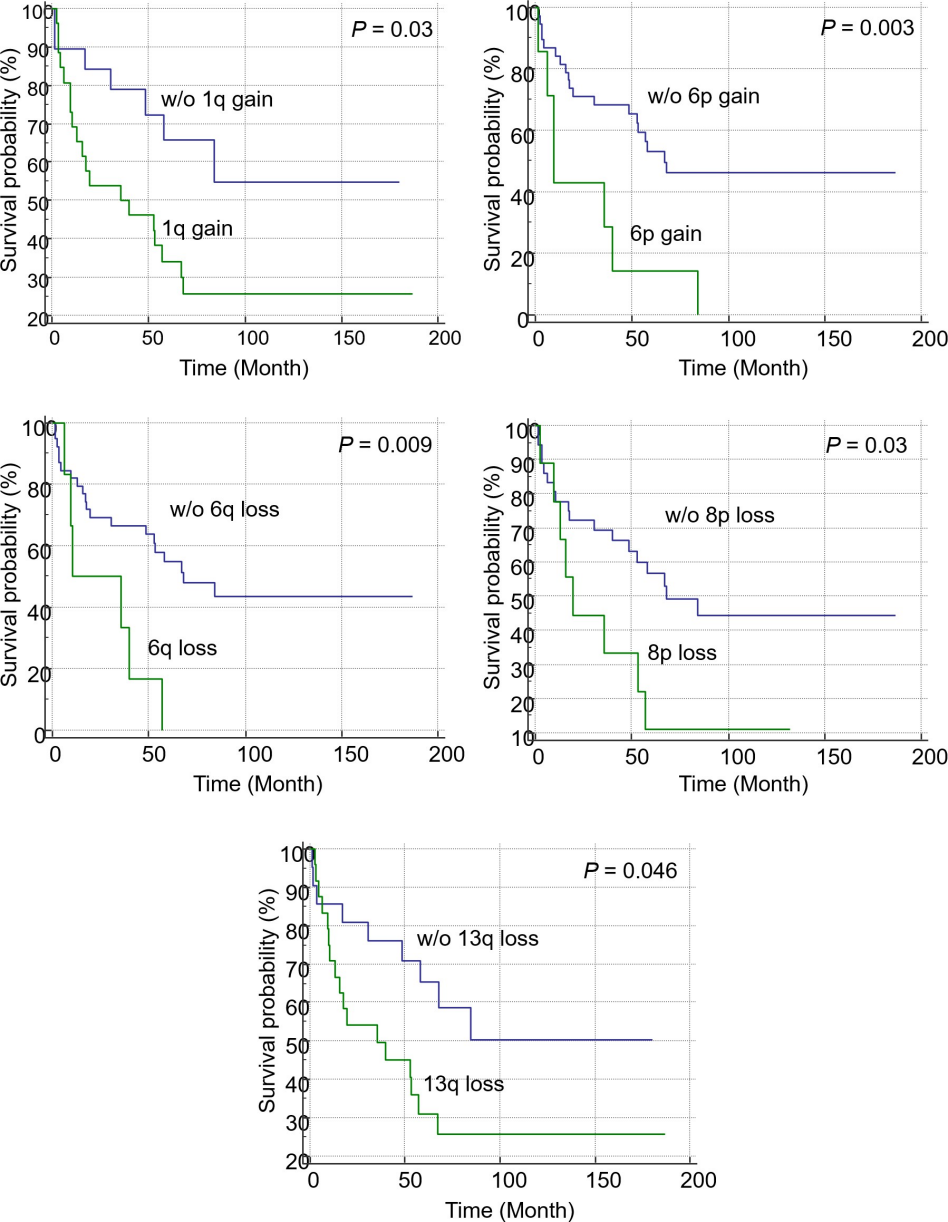
Kaplan-Meier analysis of overall survival in patients with copy number variations. Patients with 1q gain (*P* = 0.03), 6p gain (*P* = 0.003), 6q loss (*P* = 0.009), 8p loss (*P* = 0.03), and 13q loss (*P* = 0.046) were significantly correlated with poor prognosis in the log-rank test.

**Fig 5:**
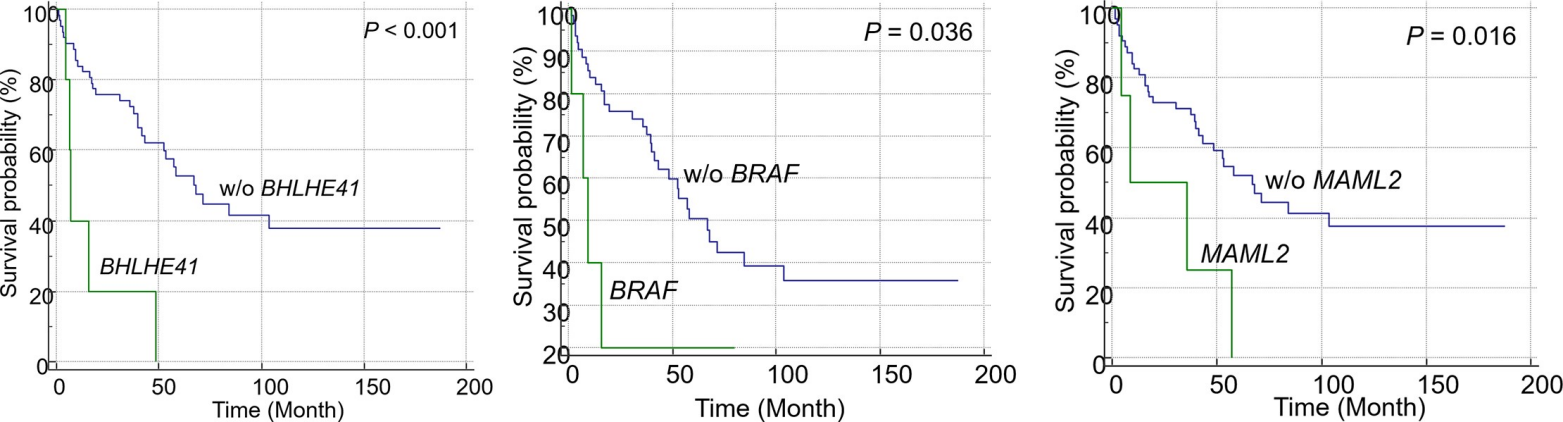
Kaplan-Meier analysis of overall survival in patients with mutations in the driver genes. *BHLHE41* (*P* < 0.001) and *MAML2* (*P* = 0.016) mutations were significantly correlated with poor prognosis in the log-rank test, while *BRAF* mutations were significantly correlated with poor prognosis only in the Breslow test.

The univariate analysis revealed that 1q gain (hazard ratio [HR] = 2.54; 95% confidence interval [CI] = 1.06–6.04; *P* = 0.036), 6p gain (HR = 3.50; 95% CI = 1.45–8.46; *P* = 0.005), 6q loss (HR = 3.33; 95% CI = 1.29–8.65; *P* = 0.013), 8p loss (HR = 2.48; 95% CI = 1.06–5.78; *P* = 0.036), and 13q loss (HR = 2.25; 95% CI = 0.99–5.08; *P* = 0.052) were associated with poor OS in patients with non-hyperdiploid MM. The multivariate analysis of these CNVs revealed that 1q gain and 6p gain were significant independent prognostic markers (Table 3). Additionally, KM analysis revealed that patients with 1q gain (*P* = 0.03; Fig 4A), 6p gain (*P* = 0.003; Fig 4B), 6q loss (*P* = 0.009; Fig 4C), 8p loss (*P* = 0.03; Fig 4D), and 13q loss (*P* = 0.046; Fig 4E) exhibited poor prognosis. In patients with driver gene mutations, univariate and multivariate analyses revealed that mutations in *MAML2* (HR = 3.32; 95% CI = 1.34–8.27; *P* = 0.010) and *BHLHE41* (HR = 5.16; 95% CI = 1.91–13.9; *P* = 0.001) were significantly associated with poor OS. The KM plot of patients with driver gene mutations is shown in Fig 5. Patients with *BHLHE41* (*P* < 0.001, Fig 5A) and *MAML2* (*P* = 0.016, Fig 5C) mutations exhibited a significantly poor prognosis in the log-rank test, while those with *BRAF* mutations exhibited a significantly poor prognosis in the Breslow test (*P* = 0.036, Fig 5B). Additionally, *CREBBP* (*P* < 0.001), *HIST1H1D* (*P* = 0.044), *PRDM1* (*P* = 0.025), *KLHL6* (*P* < 0.001), *TRAF3* (*P* = 0.049), and *UBR5* (*P* = 0.003) mutations, which were detected only in one or two patients, were significantly associated with poor prognosis.

**Table 3:** Cox proportional hazards model for factors associated with overall survival.

Variables		N	Univariable		Multivariable	
			HR (95% CI)	*P*-value	HR (95% CI)	*P*-value
CNV	1q gain	26	2.54 (1.06–6.04)	0.036	2.46 (1.02–5.92)	0.045
(Non-hyperdiploid)	6p gain	7	3.50 (1.45–8.46)	0.005	3.40 (1.38–8.38)	0.008
	6q loss	6	3.33 (1.29–8.65)	0.013		
	8p loss	9	2.48 (1.06–5.78)	0.036		
	13q loss	24	2.25 (0.99–5.08)	0.052		
Mutated driver genes	*MAML2*	4	3.41 (1.19–9.82)	0.023	3.96 (1.35–11.67)	0.013
	*BHLHE41*	5	5.16 (1.91–13.90)	0.001	6.93 (2.21–21.73)	0.001

#### Prognostic impact of CNVs identified through FISH, CG, and WES analyses

**Fig 6:**
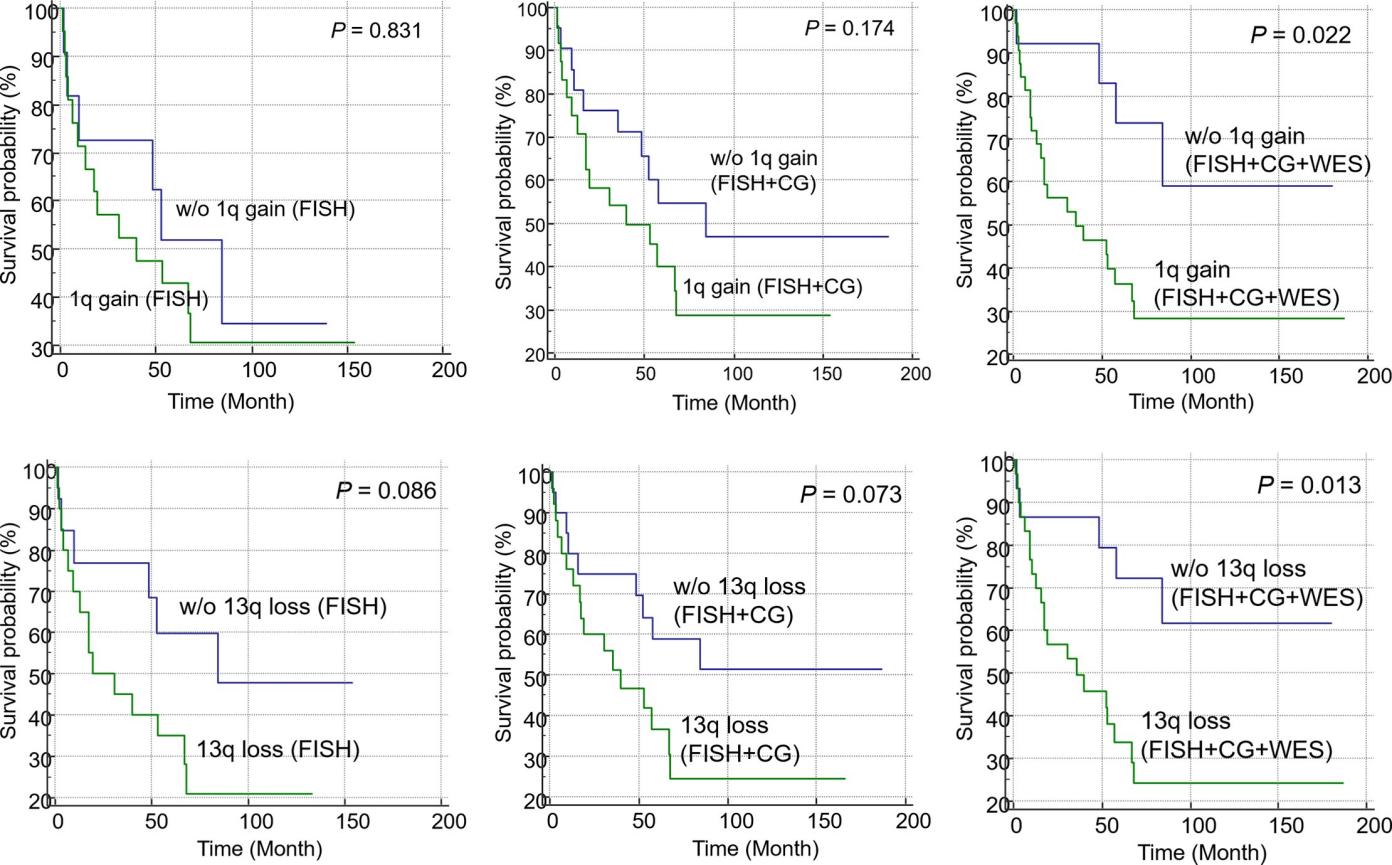
Kaplan-Meier survival curves of patients with copy number variations identified using Fluorescent in Situ Hybridization (FISH), conventional cytogenetics (CG), and Whole-Exome Sequencing (WES) analysis. Overall survival (OS) of patients with 1q gain identified through FISH+CG+WES analysis was significantly poor (C). However, the OS of patients with 1q gain identified through FISH (A) or FISH+CG (B) was not significantly different. Similarly, the OS of patients with 13q loss identified through FISH (D) and FISH+CG (E) was not significantly different. However, patients with 13q loss identified through FISH+CG+WES analysis exhibited significantly poor prognosis (F).

Quantitative results of FISH and CG are shown in Table 4. Compared with the CG analysis of metaphase chromosomes, FISH and WES analyses revealed a higher frequency of abnormalities. CNV and FISH analyses identified 1q gain in 52.2% and 60.0% of the patients, respectively. Moreover, CNV and FISH analyses identified 13q deletion in 49.3% and 48.1% of the patients, respectively. Survival analysis of patients with or without copy number alterations identified through FISH, CG, and WES analyses was performed (Fig 6). KM analysis revealed that 1q gain identified through FISH was not significantly correlated with OS (*P* = 0.831; Fig 6A). Similarly, 1q gain identified through FISH and CG analyses (FISH+CG) was not significantly associated with OS (*P* = 0.174; Fig 6B). Meanwhile, patients with 1q gain identified through WES (*P* = 0.03; Fig 4A) and FISH+CG+WES (*P* = 0.022; Fig 6C) analyses exhibited a significantly adverse OS. Furthermore, patients with 13q deletion identified through CNV exhibited poor OS (*P* = 0.046; Fig 4E). Patients with 13q deletion identified through FISH+CG+WES also exhibited poor OS (*P* = 0.013; Fig 6F). However, the genetic variations identified through FISH (*P* = 0.086; Fig 6D) and FISH+CG (*P* = 0.073; Fig 6E) were not significantly correlated with OS.

**Table 4:** Results of copy number variations and rearrangements by WES, FISH and CG in patients with NDMM.

Anomaly	WES CNV*	FISH*	CG*	FISH+CG*^□^	FISH+CG+WES*^□^
1q (1q25) gain	52.2% (35/67)	60.0% (30/50)	27.0% (17/63)	46.3% (31/67)	67.2% (45/67)
13q (13q14, RB1) deletion	49.3% (33/67)	48.1% (25/52)	34.9% (22/63)	50.7% (34/67)	58.2% (39/67)
17p (17p13, p53) deletion	7.5% (5/67)	12.5% (3/24)	1.6% (1/63)	6.5% (4/62)	9.0% (6/67)
IGH/MAF rearrangement	NA	8.0% (2/25)	3.2% (2/63)	4.8% (3/62)	NA
IGH/FGFR3 rearrangement	NA	18.5% (5/27)	1.6% (1/63)	9.7% (6/62)	NA

*In parentheses, Number of patients with positive results/Number of patients with total MM cases tested.

^□^Patients with positive on at least one of the tests were counted.

Abbreviations: WES, whole-exome sequencing; CNV, copy number variation; FISH, flourescence in situ hybridization; CG, conventional cytogenetics.

#### Mutational burden (CNVs and driver gene mutations) as a prognostic factor

**Fig 7:**
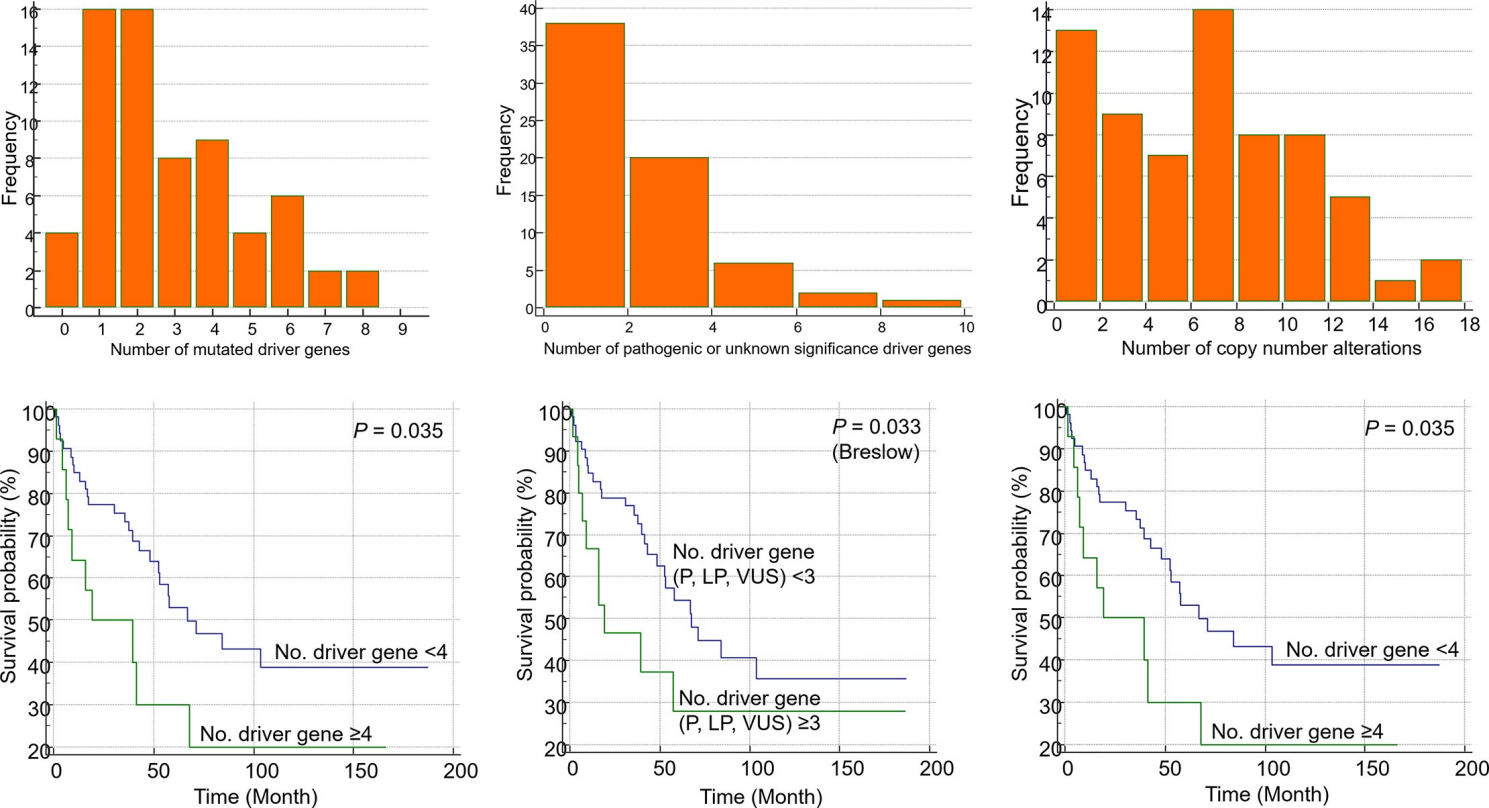
Distribution of the number of mutated driver genes in each patient. Distribution plots for the total number of (A) driver mutations, (B) selected mutations with variants of unknown significance, likely pathogenic, and pathogenic according to the ACMG guideline, and (C) copy number variations in each patient. Kaplan-Meier plots revealed that patients with (D) > 5 gene variants, (E) > 4 selected gene mutations, and (F) > 5 copy number variations exhibited poor overall survival.

The distribution of the number of mutated driver genes in each patient is shown in Fig 7A. One or more mutations were detected in 63 patients (94%). The median number of total mutations was 2.0 (95% CI = 2.0–3.0; range = 0–8). The distribution plot of only VUS or higher mutations (ACMG classification) is shown in Fig 7B. The median number of mutations was 1.0 (95% CI = 1.0–2.0; range = 0–8). The CNVs were detected in 56 patients (83.6%). The median total CNV number in each patient was 6.0 (95% CI = 5.7–7.0; range 0–16; Fig 7C).

KM analysis revealed that both CNVs and driver gene mutations were associated with poor prognosis in the group with a high mutational burden. Patients with ≥ 4 gene mutations exhibited lower OS than those with < 4 gene mutations (*P* = 0.035; Fig 7D). Additionally, patients with ≥ 3 mutant (VUS) driver genes exhibited poorer prognosis than patients with < 3 mutant driver genes (*P* = 0.033; Breslow test; Fig 7E). The survival rate of patients with ≥ 4 CNVs was lower than that of patients with < 4 CNVs *(P* = 0.035; Fig 7F).

#### Factors affecting OS in patients with hyperdiploid NDMM

**Fig 8:**
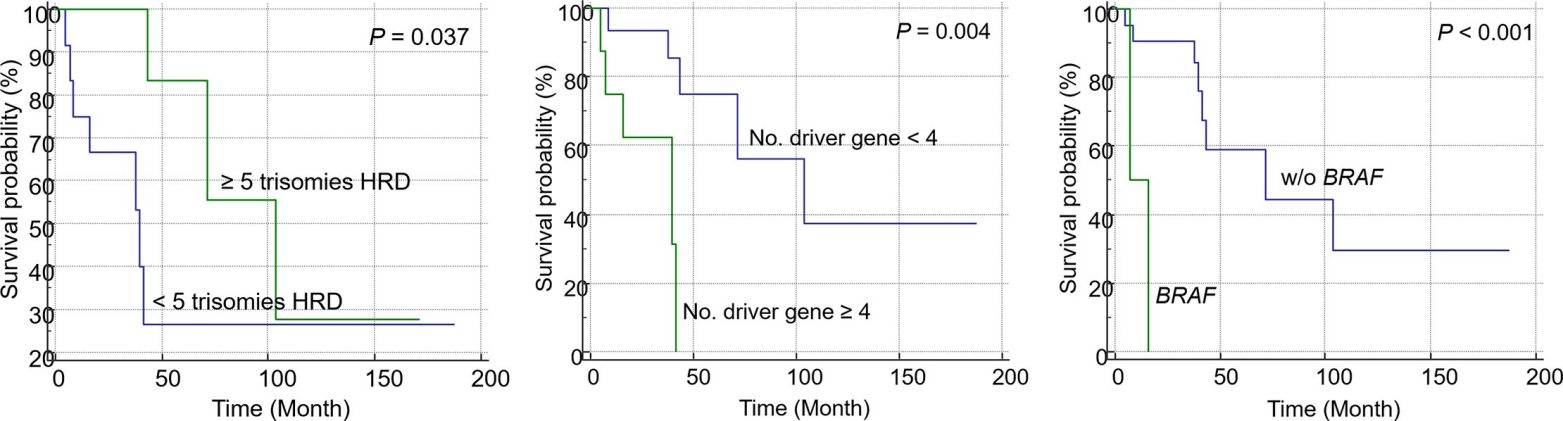
Kaplan-Meier curves showing the overall survival of patients with hyperdiploid Multiple Myeloma (MM). Patients with hyperdiploid MM were subdivided based on the (A) number of trisomies (≥ 5 vs. <5) (*P* = 0.037), (B) number of mutated driver genes (≥ 3 vs. <3) (*P* < 0.001), and (C) the presence of *BRAF* mutation (*P* < 0.001).

Among patients with hyperdiploid NDMM, patients with ≥ 5 trisomies exhibited better prognosis than those with < 5 trisomies (*P* = 0.037; Fig 8A). Patients with < 4 driver gene mutations exhibited a more favorable prognosis than those with ≥ 4 driver gene mutations (*P* = 0.004; Fig 8B). The survival analysis of patients with hyperdiploid NDMM with or without driver gene mutations revealed that patients without *BRAF* mutations exhibited a better prognosis than those with *BRAF* mutations (*P* < 0.001; Fig 8C).

#### OS of clusters classified based on K-means analysis

**Fig 9:**
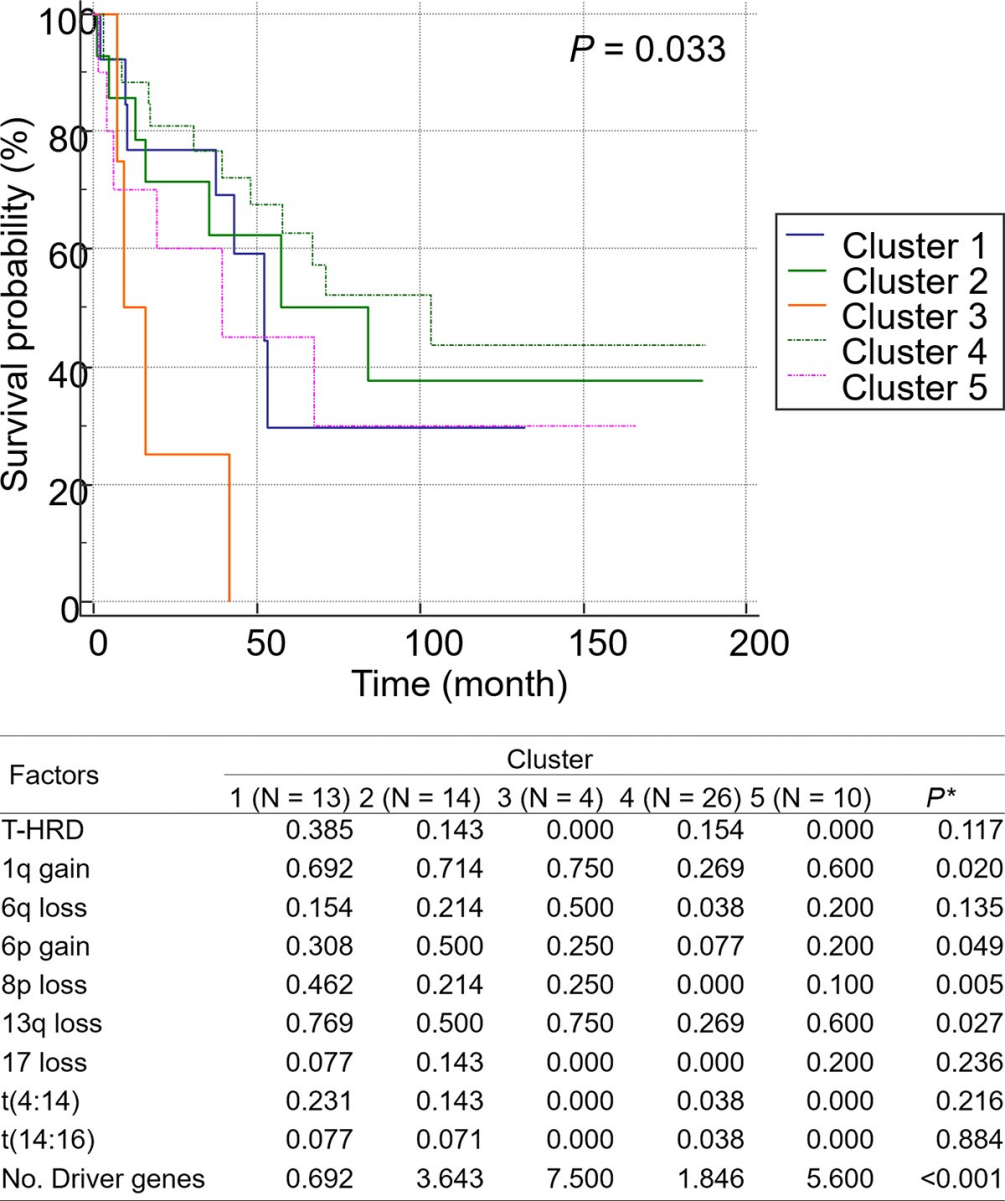
Kaplan-Meier curves for Overall Survival (OS) with subdivided driver groups. (A) The cluster 3 group exhibited significantly lower OS than clusters 1, 2, 4, and 5 (*P* = 0.019). (B) Final cluster centers for variables, including those used for K-means analysis, are classified based on each cluster. The significant clustering factors were 1q gain, 6p gain, 8p loss, 13q loss, and the number of driver gene mutations (*P* < 0.05). For example, the cluster 3 group, which exhibited the worst prognosis, had a clustering center with 1q gain, 6p gain, 13q loss, and driver gene mutation number of 7.5. *P-value was calculated using analysis of variance.

Several factors affecting the survival rate of patients with NDMM identified in this study were classified according to the K-means clustering method. The following factors were included in the analysis: True-hyperdiploid (T-HRD) (>5 trisomy), 1q gain, 6q loss, 6p gain, 8p loss, 13q loss, 17 loss, t(4:14), t(14:16), and the total number of driver gene mutations for each patient. Upon classification into five clusters, 1q gain, 6p gain, 8p loss, 13q loss, and the number of driver gene mutations were identified as the significant clustering factors. Among them, the cluster 3 group with 1q gain, 6p gain, 13q loss, and driver gene mutation number of 7.5 as the clustering center was associated with poor prognosis. In contrast, the cluster 4 group with no CNVs and a low centering value with a driver gene mutation number of 1.8 exhibited the best prognosis. The cluster 5 group with driver gene mutation number of 5.6, 1q gain, and 13q loss exhibited significantly poorer OS than the cluster 4 group (*P* = 0.033; Fig 9).

## Discussion

In this study, the CNV and somatic variant profiles of Korean patients with NDMM were identified using WES. A comprehensive analysis revealed that the gain of 1q and 6p chromosome arms and the loss of 6q, 8q, and 13q chromosome arms were associated with poor OS. Additionally, *MAML2* and *BHLHE41* mutations were shown to have an adverse prognostic impact on OS. Patients with a high frequency of CNVs or a high number of mutations exhibited poor prognosis. Cluster analysis revealed that patients with the highest number of total driver gene mutations along with 1q gain, 6p gain, and 13q loss were associated with the poorest prognosis. These findings suggested the utility of integrated analysis of CNVs and somatic mutations in predicting prognosis.

The differences in the genomic profile between different ethnic groups were examined. The genomic profile of Korean patients with NDMM was compared with that reported previously [23–26]. Among the CNVs, 1q gain (52.2%) and 13q loss (49.3%) were most frequently detected, followed by losses of 8p, 14q, 1p, and 6q. The prevalence of 6p gain and 1q gain (26–45%) in this study was higher than that reported in previous studies. The frequency of 1q gain detection using FISH was high in Korean patients with myelodysplastic syndrome [27]. This indicated that 1q gain is a candidate CNV that is specific for Korean patients with myeloma. Among the somatic variants, the frequency of mutations in the following genes was higher than 6%: *IGLL5*, *ATM*, *NRAS*, *KRAS*, *DIS3*, *MAN2C1*, *BHLHE41*, *MAML2*, and *BRAF*. The frequency of *IGLL5* mutation was high (18%), which is reported to occur exclusively with *KRAS*/*NRAS* mutation [28]. In contrast, the frequency of mutated genes in the MAPK pathway, such as *KRAS*, *NRAS*, and *BRAF*, was lower than that reported in previous studies on the Caucasian population (20–36%) [9, 25, 29, 30]. This low frequency might be owing to the characteristics of the enrolled patients. Previously, Cifola et al. reported that the frequency of *KRAS*/*NRAS* mutations was low in plasma cell leukemia, which is the aggressive and high-risk form of plasma cell dyscrasia [31]. As this study performed integrative analysis on patients with ≥ 60% plasma cells in the BM aspirate, the characteristics of patients with advanced disease could contribute to the low frequency of *KRAS*/*NRAS* mutations in this study. Meanwhile, the incidence of other mutated genes in patients with NDMM varied in different studies. This variability might result from the heterogeneous composition of patients with myeloma rather than the ethnic difference.

CNVs are considered to be one of the most important drivers of cancer development and progression [12, 24, 25, 30, 32–35]. Recently, various methods have been developed for analyzing CNV through WES, which has enabled the integrated analysis of CNVs and somatic mutations [36] in addition to overcoming the limitations of CNV detection through CG and/or FISH. In the case of NDMM, the prognostic relevance was not clearly revealed except for the well-known variants such as t(4;14) and del(17). The effects of these abnormalities, especially for 1q gain and 13q loss on prognosis are controversial, and only a few studies have included them for risk stratification criteria [37–39]. The prognostic discrepancy could be caused by method sensitivity in addition to several unmeasuring confounding factors. A previous study showed that 5–53% of patients who were normal in FISH and cytogenetics had abnormality in chromosomal microarray test [40]. CNV detection through WES can overcome the limitations associated with low proliferative activity of cells or a low number of neoplastic plasma cells in the BM when compared with CNV detection through CG. Moreover, the detection of CNVs through single nucleotide polymorphism array or WES, which can encompass the entire chromosome, would be more helpful in predicting prognosis than FISH, which only detects small target chromosomal areas. In this study, 1q gain and 13q loss identified through WES analysis adversely affected OS, whereas 1q gain and 13q loss identified through FISH and/or CG methods did not have prognostic relevance. The deletion of whole arm of 13q (N = 29; 87.9%) and the gain of whole arm of 1q (N = 30; 85.7%) were observed in a majority of patients with 13q deletion and 1q gain in this study. Previously, Binder et al. analyzed the prognosis of patients with monosomy 13 and del(13q) separately in 1,181 NDMM patients, and reported that only patients with monosomy 13 had adverse prognosis [41]. Both of these studies suggest that the 13q loss, which had previously shown neutral or favorable results, may have included many patients with interstitial deletion rather than whole chromosome loss. Furthermore, it provides additional rationale for enabling the integration of WES and FISH tests to provide enhanced genetic information for risk stratification and improved prediction of outcomes.

Additionally, WES could explore the regions that are not covered by routine FISH probes. Recently, CNVs including 6p gain, 6q loss, and 8p loss have been reported to emerging recurrent alterations in multiple myeloma [40]. As CNV detection technologies have been developed, interests in the clinical implications of these new and emerging marker have raised. In this study, patients with 6p gain, 6q loss, and 8p loss were statistically significantly associated with poor prognosis. Further studies are needed to validate these CNVs as adverse prognostic markers in a large cohort.

In this study, *BHLHE41* and *MAML2* somatic mutations were associated with poor prognosis. *BHLHE41*, which is located at 12p12.1, is known as a helix-loop-helix superfamily domain that is involved in various cellular functions, such as proliferation, differentiation, tumorigenesis, and circadian rhythms [42]. The expression of *BHLHE41* is reported to be upregulated in patients with Waldenstrom macroglobulinemia [43], and might be associated with poor prognosis in NDMM. *MAML2* is located at 11q21. The prognostic relevance of MECT1–MAML2 and CRTC1-MAML2 fusion oncogenes has been reported in mucoepidermoid carcinomas [44, 45]. In this study, *MAML2* mutations exhibited positive correlation with various CNVs including 17q loss and 22q loss, and these variations may aid in predicting the prognosis. *TP53* mutations are a well-known adverse prognostic factor in myeloma. Niccolo et al. demonstrated that *TP53* mutations were associated with adverse progression-free survival (PFS) and OS and that a rare mutation in *DNAH11* affected OS [9]. Another study reported that *TP53*, *ATR*, *ATM*, and *ZFHX4* mutations are associated with poor PFS or OS [30]. Although TP53 did not show statistical significance in OS in this study (P = 0.613), it is presumed to be due to too small number of mutation-positive patients (N = 3). However, it could be owing to the differences in inclusion criteria, such as selecting only patients with >60% PC or targeting the Korean population. Most previous studies report the heterogeneous mutational landscape of MM. Some patients exhibited redundancy in gene mutations as two or more mutations were detected in genes involved in the same pathway [12, 46, 47]. Consistent with these findings the current study suggested the heterogeneity of genomic variants in MM. We suggest that the prediction power of mutational burden in structural and somatic variants is higher than that of a single variation.

In this study, we presented novel prognostic subgroups in patients with NDMM through K-means clustering analysis. The clusters were divided according to CNVs and mutations that affect OS. The “Cluster 3” exhibited the highest number of driver gene mutations and CNVs, including 1q gain, 13q loss, and 6q loss. Although efforts to stratify potential subgroups according to OS were attempted previously [9, 10], the characteristics of each cluster group have not been distinguished. However, this study revealed that prognosis-related CNVs, such as 1q gain, 13q loss, and 6q loss have clustering relevance, whereas somatic gene mutations have no clustering importance. The overall number of mutations in the driver gene panel played a significant role in stratifying each group.

Recently, prognosis-related classifications are being reconsidered for patients with hyperdiploid MM [48, 49]. In this study, patients with hyperdiploid MM and more than five trisomies exhibited favorable OS, which was based on a review of hyperdiploid variations that affected prognosis. Moreover, patients with hyperdiploid MM and an increased number of driver gene mutations exhibited poor prognosis. These results suggest that the integrated analysis of mutation burden (both CNVs and somatic mutations) using WES could aid in precisely predicting the clinical outcomes of patients with MM.

This study has several limitations; CD138 purification and sorting steps have not been implemented. Instead, only patients with >60% plasma cells in the BM aspirate were selected for the study. Most patients showed high numbers of plasma cells in the BM. Additionally, there were no control samples to remove the germline background. To overcome this limitation, the gene variants observed in healthy Korean individuals (n = 2,000) were removed. And, we focused on the driver genes selected by multiple algorithms in several previous studies. Based on the ACMG guidelines, variants with VUS, pathogenic, and likely pathogenic were separately analyzed. Furthermore, CNVs under 2,500 kb were excluded in CNV analysis. However, this study demonstrated that large CNVs with size > 2,500 kb were poor prognostic factors. As CNV analysis cannot detect chromosomal translocations, the results of translocations, such as t(4;14) and t(14;16) were referenced for clustering integrated analysis.

In conclusion, this study comprehensively analyzed the somatic mutations and CNVs of patients with NDMM using WES. Additionally, this study proposed a new method for classifying patient groups with poor prognosis and predicting OS. MM is a heterogeneous disease comprising several subclones, and multiple driver gene mutations are detected in one patient. Therefore, an integrated analysis through the application of WES in the future would aid in predicting the prognosis in a clinical setting.

## Supporting information

S1 File (DOCX)

## References

[1] Hong J, Lee JH. Recent advances in multiple myeloma: a Korean perspective. Korean J Intern Med. 2016;31(5):820–34. 10.3904/kjim.2015.408 27604794 PMC5016289

[2] Palumbo A, Anderson K. Multiple myeloma. The New England journal of medicine. 2011;364(11):1046–60. 10.1056/NEJMra1011442 21410373

[3] Morgan GJ, Walker BA, Davies FE. The genetic architecture of multiple myeloma. Nature reviews Cancer. 2012;12(5):335–48. 10.1038/nrc3257 22495321

[4] Kumar SK, Rajkumar SV, Dispenzieri A, Lacy MQ, Hayman SR, Buadi FK, et al Improved survival in multiple myeloma and the impact of novel therapies. Blood. 2008;111(5):2516–20. 10.1182/blood-2007-10-116129 17975015 PMC2254544

[5] Kumar SK, Dispenzieri A, Lacy MQ, Gertz MA, Buadi FK, Pandey S, et al Continued improvement in survival in multiple myeloma: changes in early mortality and outcomes in older patients. Leukemia. 2014;28(5):1122–8. 10.1038/leu.2013.313 24157580 PMC4000285

[6] Avet-Loiseau H, Durie BG, Cavo M, Attal M, Gutierrez N, Haessler J, et al Combining fluorescent in situ hybridization data with ISS staging improves risk assessment in myeloma: an International Myeloma Working Group collaborative project. Leukemia. 2013;27(3):711–7. 10.1038/leu.2012.282 23032723 PMC3972006

[7] Palumbo A, Avet-Loiseau H, Oliva S, Lokhorst HM, Goldschmidt H, Rosinol L, et al Revised International Staging System for Multiple Myeloma: A Report From International Myeloma Working Group. Journal of clinical oncology: official journal of the American Society of Clinical Oncology. 2015;33(26):2863–9. 10.1200/JCO.2015.61.2267 26240224 PMC4846284

[8] Maura F, Bolli N, Angelopoulos N, Dawson KJ, Leongamornlert D, Martincorena I, et al Genomic landscape and chronological reconstruction of driver events in multiple myeloma. Nature communications. 2019;10(1):3835 10.1038/s41467-019-11680-1 31444325 PMC6707220

[9] Bolli N, Biancon G, Moarii M, Gimondi S, Li Y, de Philippis C, et al Analysis of the genomic landscape of multiple myeloma highlights novel prognostic markers and disease subgroups. Leukemia. 2018;32(12):2604–16. 10.1038/s41375-018-0037-9 29789651 PMC6092251

[10] Walker BA, Mavrommatis K, Wardell CP, Ashby TC, Bauer M, Davies FE, et al Identification of novel mutational drivers reveals oncogene dependencies in multiple myeloma. 2018;132(6):587–97. 10.1182/blood-2018-03-840132 PMC6097138 29884741

[11] Walker BA. Whole Exome Sequencing in Multiple Myeloma to Identify Somatic Single Nucleotide Variants and Key Translocations Involving Immunoglobulin Loci and MYC. Methods in molecular biology (Clifton, NJ). 2018;1792:71–95. 10.1007/978-1-4939-7865-6_6 29797253

[12] Robiou du Pont S, Cleynen A, Fontan C, Attal M, Munshi N, Corre J, et al Genomics of Multiple Myeloma. Journal of clinical oncology: official journal of the American Society of Clinical Oncology. 2017;35(9):963–7. 10.1200/JCO.2016.70.6705 28297630

[13] Rajkumar SV, Dimopoulos MA, Palumbo A, Blade J, Merlini G, Mateos MV, et al International Myeloma Working Group updated criteria for the diagnosis of multiple myeloma. The Lancet Oncology. 2014;15(12):e538–48. 10.1016/S1470-2045(14)70442-5 25439696

[14] Li H, Durbin R. Fast and accurate short read alignment with Burrows-Wheeler transform. Bioinformatics (Oxford, England). 2009;25(14):1754–60. 10.1093/bioinformatics/btp324 19451168 PMC2705234

[15] McKenna A, Hanna M, Banks E, Sivachenko A, Cibulskis K, Kernytsky A, et al The Genome Analysis Toolkit: a MapReduce framework for analyzing next-generation DNA sequencing data. Genome research. 2010;20(9):1297–303. 10.1101/gr.107524.110 20644199 PMC2928508

[16] Ng PC, Henikoff S. SIFT: Predicting amino acid changes that affect protein function. Nucleic acids research. 2003;31(13):3812–4. 10.1093/nar/gkg509 12824425 PMC168916

[17] Kumar P, Henikoff S, Ng PC. Predicting the effects of coding non-synonymous variants on protein function using the SIFT algorithm. Nature protocols. 2009;4(7):1073–81. 10.1038/nprot.2009.86 19561590

[18] Kircher M, Witten DM, Jain P, O’Roak BJ, Cooper GM, Shendure J. A general framework for estimating the relative pathogenicity of human genetic variants. Nature genetics. 2014;46(3):310–5. 10.1038/ng.2892 24487276 PMC3992975

[19] Adzhubei IA, Schmidt S, Peshkin L, Ramensky VE, Gerasimova A, Bork P, et al A method and server for predicting damaging missense mutations. Nature methods. 2010;7(4):248–9. 10.1038/nmeth0410-248 20354512 PMC2855889

[20] Richards S, Aziz N, Bale S, Bick D, Das S, Gastier-Foster J, et al Standards and guidelines for the interpretation of sequence variants: a joint consensus recommendation of the American College of Medical Genetics and Genomics and the Association for Molecular Pathology. Genetics in medicine: official journal of the American College of Medical Genetics. 2015;17(5):405–24. 10.1038/gim.2015.30 25741868 PMC4544753

[21] Talevich E, Shain AH, Botton T, Bastian BC. CNVkit: Genome-Wide Copy Number Detection and Visualization from Targeted DNA Sequencing. PLoS computational biology. 2016;12(4):e1004873 10.1371/journal.pcbi.1004873 27100738 PMC4839673

[22] Olshen AB, Venkatraman ES, Lucito R, Wigler M. Circular binary segmentation for the analysis of array-based DNA copy number data. Biostatistics (Oxford, England). 2004;5(4):557–72. 10.1093/biostatistics/kxh008 15475419

[23] Walker BA, Leone PE, Jenner MW, Li C, Gonzalez D, Johnson DC, et al Integration of global SNP-based mapping and expression arrays reveals key regions, mechanisms, and genes important in the pathogenesis of multiple myeloma. Blood. 2006;108(5):1733–43. 10.1182/blood-2006-02-005496 16705090

[24] Avet-Loiseau H, Li C, Magrangeas F, Gouraud W, Charbonnel C, Harousseau JL, et al Prognostic significance of copy-number alterations in multiple myeloma. Journal of clinical oncology: official journal of the American Society of Clinical Oncology. 2009;27(27):4585–90. 10.1200/JCO.2008.20.6136 19687334 PMC2754906

[25] Manier S, Salem KZ, Park J, Landau DA, Getz G, Ghobrial IM. Genomic complexity of multiple myeloma and its clinical implications. Nature reviews Clinical oncology. 2017;14(2):100–13. 10.1038/nrclinonc.2016.122 27531699

[26] Shah V, Sherborne AL, Walker BA, Johnson DC, Boyle EM, Ellis S, et al Prediction of outcome in newly diagnosed myeloma: a meta-analysis of the molecular profiles of 1905 trial patients. Leukemia. 2018;32(1):102–10. 10.1038/leu.2017.179 28584253 PMC5590713

[27] Lee DS, Kim SH, Seo EJ, Park CJ, Chi HS, Ko EK, et al Predominance of trisomy 1q in myelodysplastic syndromes in Korea: is there an ethnic difference? A 3-year multi-center study. Cancer genetics and cytogenetics. 2002;132(2):97–101. 10.1016/s0165-4608(01)00533-7 11850068

[28] White BS, Lanc I, O’Neal J, Gupta H, Fulton RS, Schmidt H, et al A multiple myeloma-specific capture sequencing platform discovers novel translocations and frequent, risk-associated point mutations in IGLL5. Blood cancer journal. 2018;8(3):35 10.1038/s41408-018-0062-y 29563506 PMC5862875

[29] Hu Y, Chen W, Wang J. Progress in the identification of gene mutations involved in multiple myeloma. OncoTargets and therapy. 2019;12:4075–80. 10.2147/OTT.S205922 31213829 PMC6538831

[30] Walker BA, Boyle EM, Wardell CP, Murison A, Begum DB, Dahir NM, et al Mutational Spectrum, Copy Number Changes, and Outcome: Results of a Sequencing Study of Patients With Newly Diagnosed Myeloma. Journal of clinical oncology: official journal of the American Society of Clinical Oncology. 2015;33(33):3911–20. 26282654 10.1200/JCO.2014.59.1503 PMC6485456

[31] Cifola I, Lionetti M, Pinatel E, Todoerti K, Mangano E, Pietrelli A, et al Whole-exome sequencing of primary plasma cell leukemia discloses heterogeneous mutational patterns. Oncotarget. 2015;6(19):17543–58. 10.18632/oncotarget.4028 26046463 PMC4627327

[32] Prideaux SM, Conway O’Brien E, Chevassut TJ. The Genetic Architecture of Multiple Myeloma. Advances in Hematology. 2014;2014:864058 10.1155/2014/864058 24803933 PMC3996928

[33] Walker BA, Leone PE, Chiecchio L, Dickens NJ, Jenner MW, Boyd KD, et al A compendium of myeloma-associated chromosomal copy number abnormalities and their prognostic value. Blood. 2010;116(15):e56–65. 10.1182/blood-2010-04-279596 20616218

[34] Kim M, Ju YS, Lee EJ, Kang HJ, Kim HS, Cho HC, et al Abnormalities in Chromosomes 1q and 13 Independently Correlate With Factors of Poor Prognosis in Multiple Myeloma. Annals of laboratory medicine. 2016;36(6):573–82. 10.3343/alm.2016.36.6.573 27578511 PMC5011111

[35] Kim M, Lee SH, Kim J, Lee SE, Kim YJ, Min CK. Copy number variations could predict the outcome of bortezomib plus melphalan and prednisone for initial treatment of multiple myeloma. Genes, chromosomes & cancer. 2015;54(1):20–7. 10.1002/gcc.22213 25145975

[36] Marchuk DS, Crooks K, Strande N, Kaiser-Rogers K, Milko LV, Brandt A, et al Increasing the diagnostic yield of exome sequencing by copy number variant analysis. PloS one. 2018;13(12):e0209185 10.1371/journal.pone.0209185 30557390 PMC6296659

[37] Mikhael JR, Dingli D, Roy V, Reeder CB, Buadi FK, Hayman SR, et al Management of newly diagnosed symptomatic multiple myeloma: updated Mayo Stratification of Myeloma and Risk-Adapted Therapy (mSMART) consensus guidelines 2013. Mayo Clinic proceedings. 2013;88(4):360–76. 10.1016/j.mayocp.2013.01.019 23541011

[38] Sonneveld P, Avet-Loiseau H, Lonial S, Usmani S, Siegel D, Anderson KC, et al Treatment of multiple myeloma with high-risk cytogenetics: a consensus of the International Myeloma Working Group. Blood. 2016;127(24):2955–62. 10.1182/blood-2016-01-631200 27002115 PMC4920674

[39] Munshi NC, Anderson KC, Bergsagel PL, Shaughnessy J, Palumbo A, Durie B, et al Consensus recommendations for risk stratification in multiple myeloma: report of the International Myeloma Workshop Consensus Panel 2. Blood. 2011;117(18):4696–700. 10.1182/blood-2010-10-300970 21292777 PMC3293763

[40] Pugh TJ, Fink JM, Lu X, Mathew S, Murata-Collins J, Willem P, et al Assessing genome-wide copy number aberrations and copy-neutral loss-of-heterozygosity as best practice: An evidence-based review from the Cancer Genomics Consortium working group for plasma cell disorders. Cancer genetics. 2018;228–229:184–96. 10.1016/j.cancergen.2018.07.002 30393007

[41] Binder M, Rajkumar SV, Ketterling RP, Greipp PT, Dispenzieri A, Lacy MQ, et al Prognostic implications of abnormalities of chromosome 13 and the presence of multiple cytogenetic high-risk abnormalities in newly diagnosed multiple myeloma. Blood cancer journal. 2017;7(9):e600 10.1038/bcj.2017.83 28862698 PMC5709752

[42] Shen Z, Zhu L, Zhang C, Cui X, Lu J. Overexpression of BHLHE41, correlated with DNA hypomethylation in 3’UTR region, promotes the growth of human clear cell renal cell carcinoma. Oncology reports. 2019;41(4):2137–47. 10.3892/or.2019.7004 30816499 PMC6412400

[43] Trojani A, Greco A, Tedeschi A, Lodola M, Di Camillo B, Ricci F, et al Microarray demonstrates different gene expression profiling signatures between Waldenström macroglobulinemia and IgM monoclonal gammopathy of undetermined significance. Clinical lymphoma, myeloma & leukemia. 2013;13(2):208–10. 10.1016/j.clml.2013.02.012 23477935

[44] Behboudi A, Enlund F, Winnes M, Andrén Y, Nordkvist A, Leivo I, et al Molecular classification of mucoepidermoid carcinomas-prognostic significance of the MECT1-MAML2 fusion oncogene. Genes, chromosomes & cancer. 2006;45(5):470–81. 10.1002/gcc.20306 16444749

[45] Anzick SL, Chen WD, Park Y, Meltzer P, Bell D, El-Naggar AK, et al Unfavorable prognosis of CRTC1-MAML2 positive mucoepidermoid tumors with CDKN2A deletions. Genes, chromosomes & cancer. 2010;49(1):59–69. 10.1002/gcc.20719 19827123 PMC2783528

[46] Leich E, Weissbach S, Klein HU, Grieb T, Pischimarov J, Stuhmer T, et al Multiple myeloma is affected by multiple and heterogeneous somatic mutations in adhesion- and receptor tyrosine kinase signaling molecules. Blood cancer journal. 2013;3:e102 10.1038/bcj.2012.47 23396385 PMC3584721

[47] Bolli N, Avet-Loiseau H, Wedge DC, Van Loo P, Alexandrov LB, Martincorena I, et al Heterogeneity of genomic evolution and mutational profiles in multiple myeloma. Nature communications. 2014;5:2997 10.1038/ncomms3997 24429703 PMC3905727

[48] Chretien M-L, Corre J, Lauwers-Cances V, Magrangeas F, Cleynen A, Yon E, et al Understanding the role of hyperdiploidy in myeloma prognosis: which trisomies really matter? Blood. 2015;126(25):2713–9. 10.1182/blood-2015-06-650242 26516228 PMC4683332

[49] Barilà G, Bonaldi L, Grassi A, Martines A, Liço A, Macrì N, et al Identification of the true hyperdiploid multiple myeloma subset by combining conventional karyotyping and FISH analysis. Blood cancer journal. 2020;10(2):18 10.1038/s41408-020-0285-6 32066724 PMC7026173

