# Supplementary material for: Prognostic value of integrated cytogenetic, somatic variation, and copy number variation analyses in Korean patients with newly diagnosed multiple myeloma
Source: PLoS One. 2021 Feb 5;16(2):e0246322. doi: 10.1371/journal.pone.0246322 (PMC7864461; doi:10.1371/journal.pone.0246322)
Supplement: S1 File — (DOCX) [file pone.0246322.s001.docx]

**S1 Table. List of driver genes including the study**

(DOCX)

**S2 Table. Frequency of chromosome gain and loss including cytoband information** (DOCX)

**S1 Fig. Examples of copy number alteration in one patient.** Patient #1 showed hemizygous deletion of 1p, 8q, 12p, 13q, 14q, and 21q, and duplication of chromosome 3. Log R ratio (upper) and B allele frequency (lower) were described on each chromosome.

(TIF)

**S2 Fig. Diagrams for mutations for most frequent genes in NDMM patients.** (A)-(G) is diagrams for *BRAF, BHLHE41, KRAS, NRAS, IGLL5, ATM,* and *MAML2* respectively. In the case of the *BRAF* gene, p.V600E occurred in 3 patients, and KRAS showed p.Q61R(L) in 3 patients. In the case of NRAS, p.G12D(V) and p.Q61L(K) were confirmed in each of 2 patients. In the ATM, mutations such as p.S403L, p.L581V, and p.Q584X occurred.

(TIF)

**S1 Table. List of driver genes including the study**

| **No** | **Driver gene selected for study** | **Driver genes detected in this study (among selected genes)** |
| --- | --- | --- |
| 1 | ABCF1 | ABCF1 |
| 2 | ACTG1 | ARID1A |
| 3 | ARID1A | ATM |
| 4 | ARID2 | ATRX |
| 5 | ATM | BHLHE41 |
| 6 | ATRX | BRAF |
| 7 | BCL7A | C8orf34 |
| 8 | BHLHE41 | CREBBP |
| 9 | BRAF | CYLD |
| 10 | BTG1 | DIS3 |
| 11 | C8ORF34 | DNMT3A |
| 12 | CCND1 | DTX1 |
| 13 | CDKN1B | DUSP2 |
| 14 | CDKN2C | EGR1 |
| 15 | CREBBP | EP300 |
| 16 | CYLD | FAM46C |
| 17 | DIS3 | FGFR3 |
| 18 | DNMT3A | HIST1H1B |
| 19 | DTX1 | HIST1H1D |
| 20 | DUSP2 | HIST1H1E |
| 21 | EGR1 | HUWE1 |
| 22 | EP300 | IDH1 |
| 23 | FAM46C | IDH2 |
| 24 | FGFR3 | IGLL5 |
| 25 | FUBP1 | IRF1 |
| 26 | HIST1H1B | IRF4 |
| 27 | HIST1H1D | KDM6A |
| 28 | HIST1H1E | KLHL6 |
| 29 | HIST1H2BK | KMT2C |
| 30 | HUWE1 | KRAS |
| 31 | IDH1 | LTB |
| 32 | IDH2 | MAFB |
| 33 | IGLL5 | MAML2 |
| 34 | IRF1 | MAN2C1 |
| 35 | IRF4 | NCOR1 |
| 36 | KDM5C | NF1 |
| 37 | KDM6A | NFKB2 |
| 38 | KLHL6 | NFKBIA |
| 39 | KMT2B | NRAS |
| 40 | KMT2C | PABPC1 |
| 41 | KRAS | PIM1 |
| 42 | LCE1D | POT1 |
| 43 | LTB | PRDM1 |
| 44 | MAF | PRKD2 |
| 45 | MAFB | PTPN11 |
| 46 | MAML2 | RB1 |
| 47 | MAN2C1 | RFTN1 |
| 48 | MAX | RPL10 |
| 49 | NCOR1 | SAMHD1 |
| 50 | NF1 | SETD2 |
| 51 | NFKB | SF3B1 |
| 52 | NFKB2 | TBC1D29 |
| 53 | NFKBIA | TET2 |
| 54 | NRAS | TP53 |
| 55 | PABPC1 | TRAF2 |
| 56 | PIK3CA | TRAF3 |
| 57 | PIM1 | UBR5 |
| 58 | POT1 | ZNF292 |
| 59 | PRDM1 |  |
| 60 | PRKD2 |  |
| 61 | PTPN11 |  |
| 62 | RASA2 |  |
| 63 | RB1 |  |
| 64 | RFTN1 |  |
| 65 | RPL10 |  |
| 66 | RPL5 |  |
| 67 | RPRD1B |  |
| 68 | RPS3A |  |
| 69 | SAMHD1 |  |
| 70 | SETD2 |  |
| 71 | SF3B1 |  |
| 72 | SP140 |  |
| 73 | TBC1D29 |  |
| 74 | TCL1A |  |
| 75 | TET2 |  |
| 76 | TGDS |  |
| 77 | TP53 |  |
| 78 | TRAF2 |  |
| 79 | TRAF3 |  |
| 80 | UBR5 |  |
| 81 | XBP1 |  |
| 82 | ZFP36L1 |  |
| 83 | ZNF292 |  |

**S2 Table. Frequency of chromosome gain and loss including cytoband information**

1. 1q gain

| **Region** | **Number** | **Percentage** |
| --- | --- | --- |
| q21.1-q23.2 | 1 | 2.9% |
| q21.1-q23.3 | 1 | 2.9% |
| q21.1-q41 | 1 | 2.9% |
| q21.1-q44 | 23 | 65.7% |
| q21.2-q44 | 7 | 20.0% |
| q23.2-q41 | 1 | 2.9% |
| q31.3-q44 | 1 | 2.9% |
| Total | 35 | 100.0% |

1. 6p gain

| **Region** | **Number** | **Percentage** |
| --- | --- | --- |
| Whole p arm | 6 | 42.9% |
| Whole p, q arms | 4 | 28.6% |
| p21.3 | 1 | 7.1% |
| p25.3-p12.3 | 1 | 7.1% |
| p25.3-p21.1 | 1 | 7.1% |
| p25.3-p21.2 | 1 | 7.1% |
| Total | 14 | 100.0% |

1. 13q loss

| **Region** | **Number** | **Percentage** |
| --- | --- | --- |
| q11-q34 | 29 | 87.9 |
| q12.1-21.3 | 1 | 3.0% |
| q12.1-q22.1 | 1 | 3.0% |
| q13.3-q14.2 | 1 | 3.0% |
| q33.1 - q34 | 1 | 3.0% |
| Total | 33 | 100.0% |


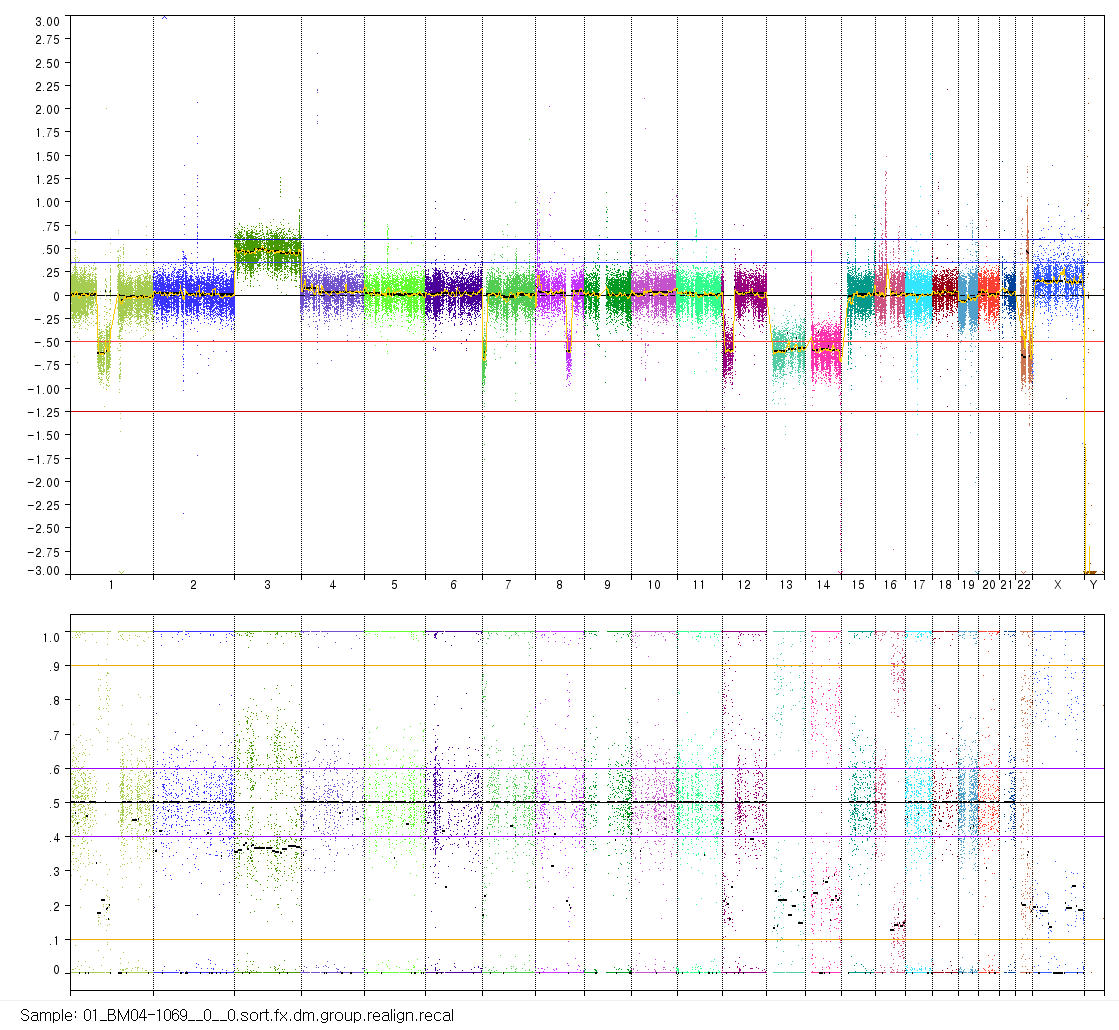


**S1 Fig.** Examples of copy number alteration in one patient. Patient #1 showed hemizygous deletion of 1p, 8q, 12p, 13q, 14q, and 21q, and duplication of chromosome 3. Log R ratio (upper) and B allele frequency (lower) were described on each chromosome.

**
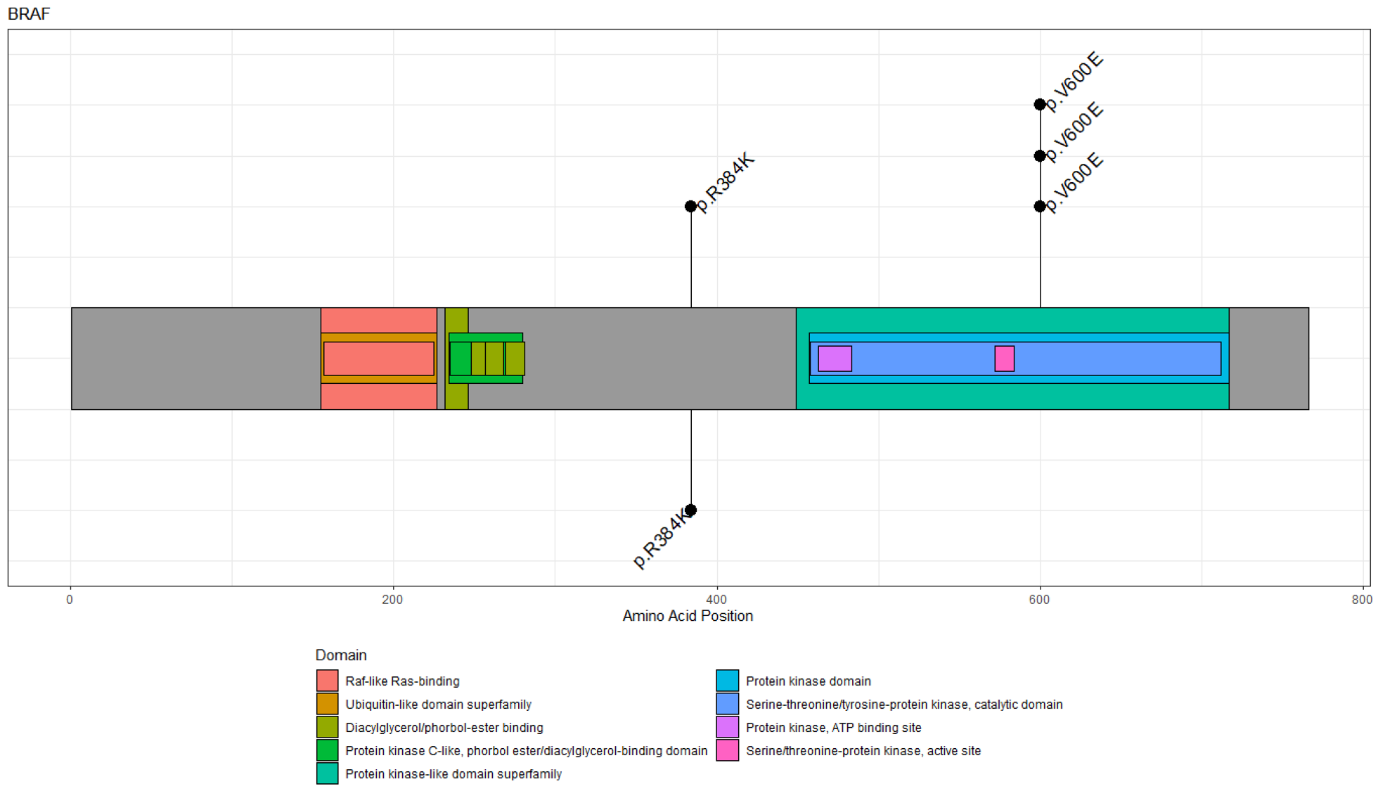
**(A)

**
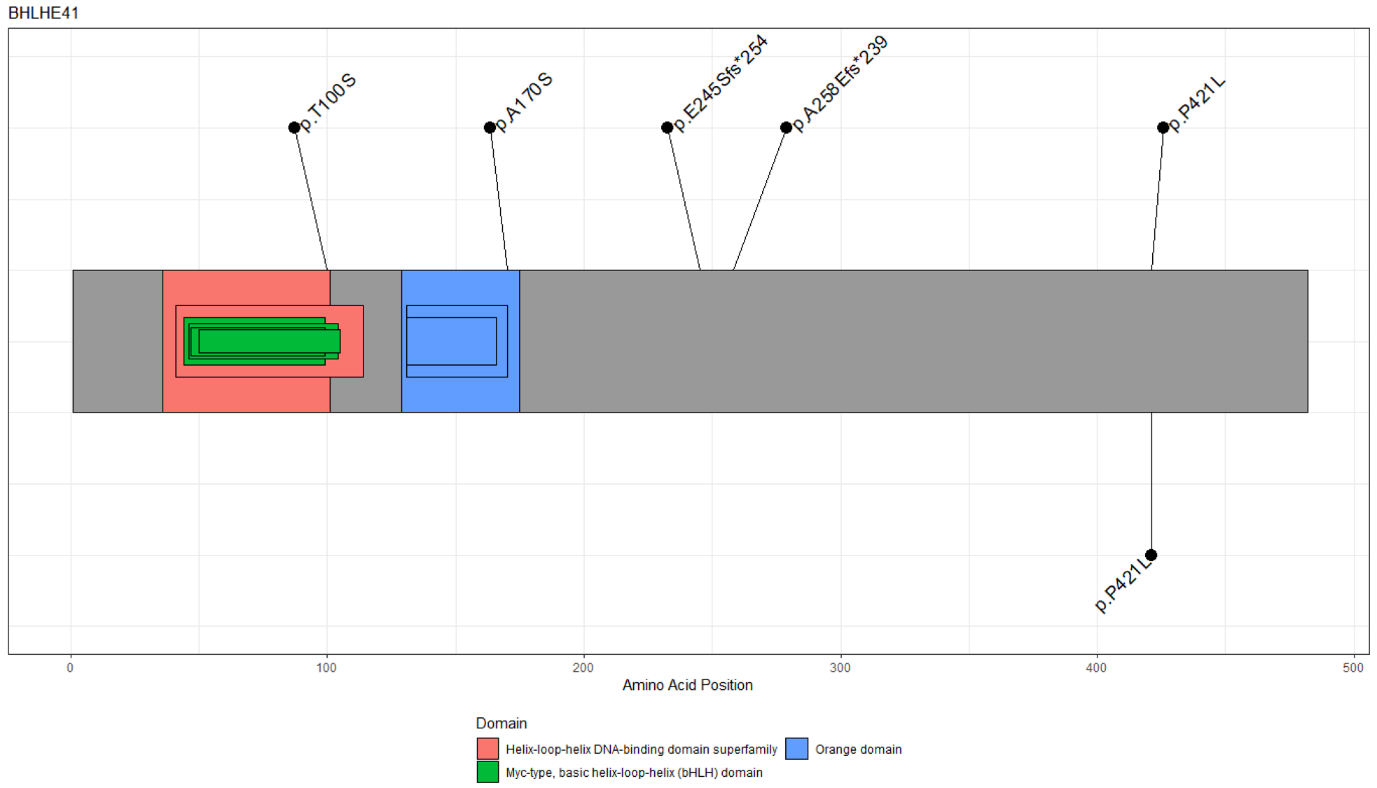
**(B)

(C)
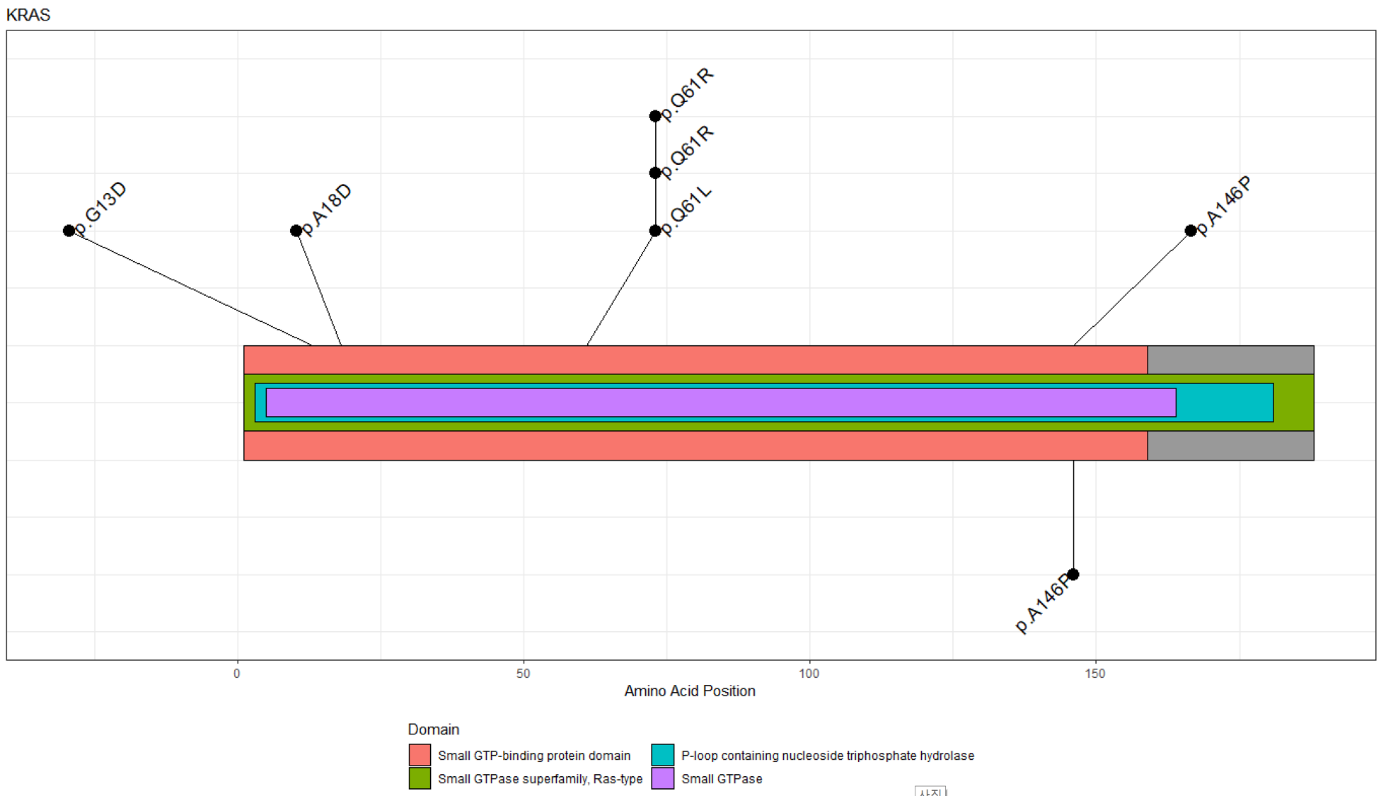


**
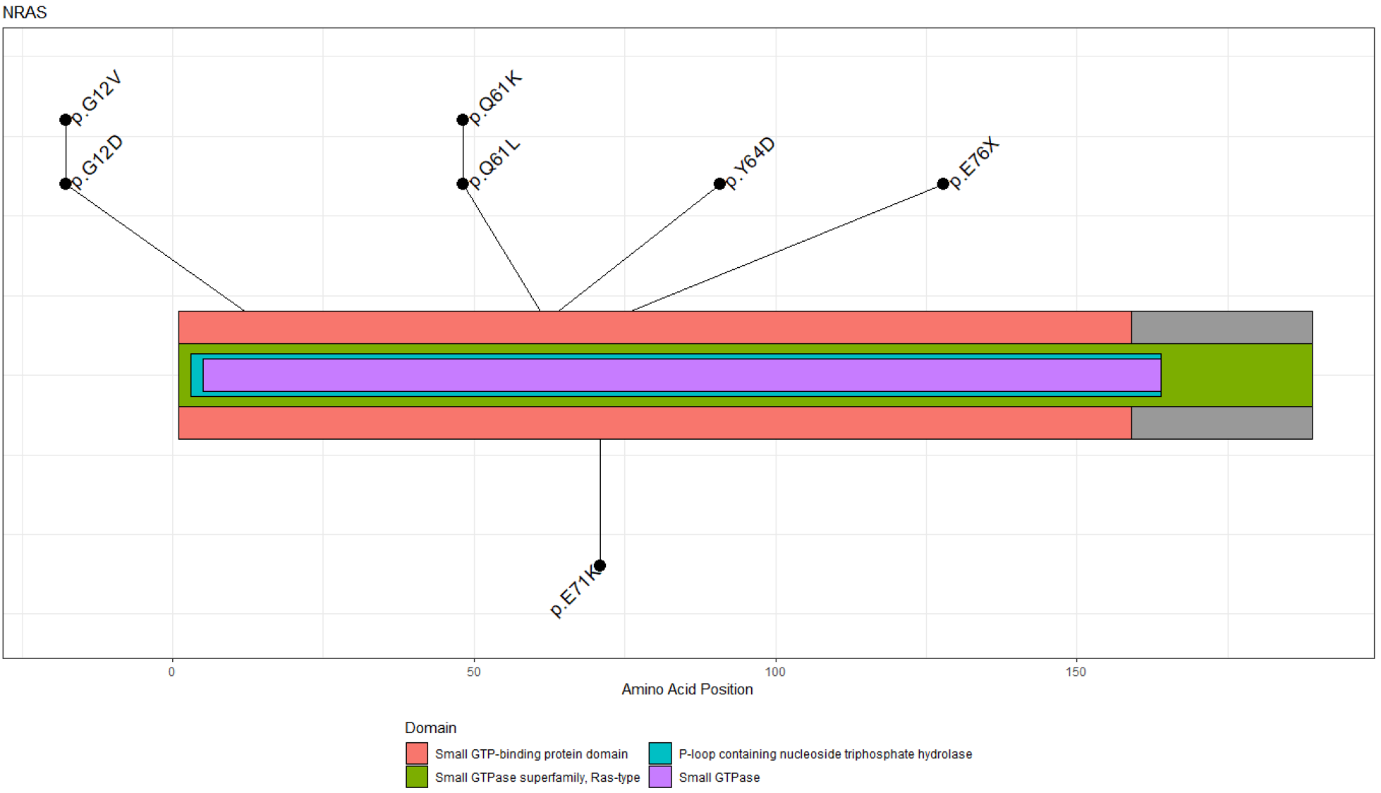
**(D)

**
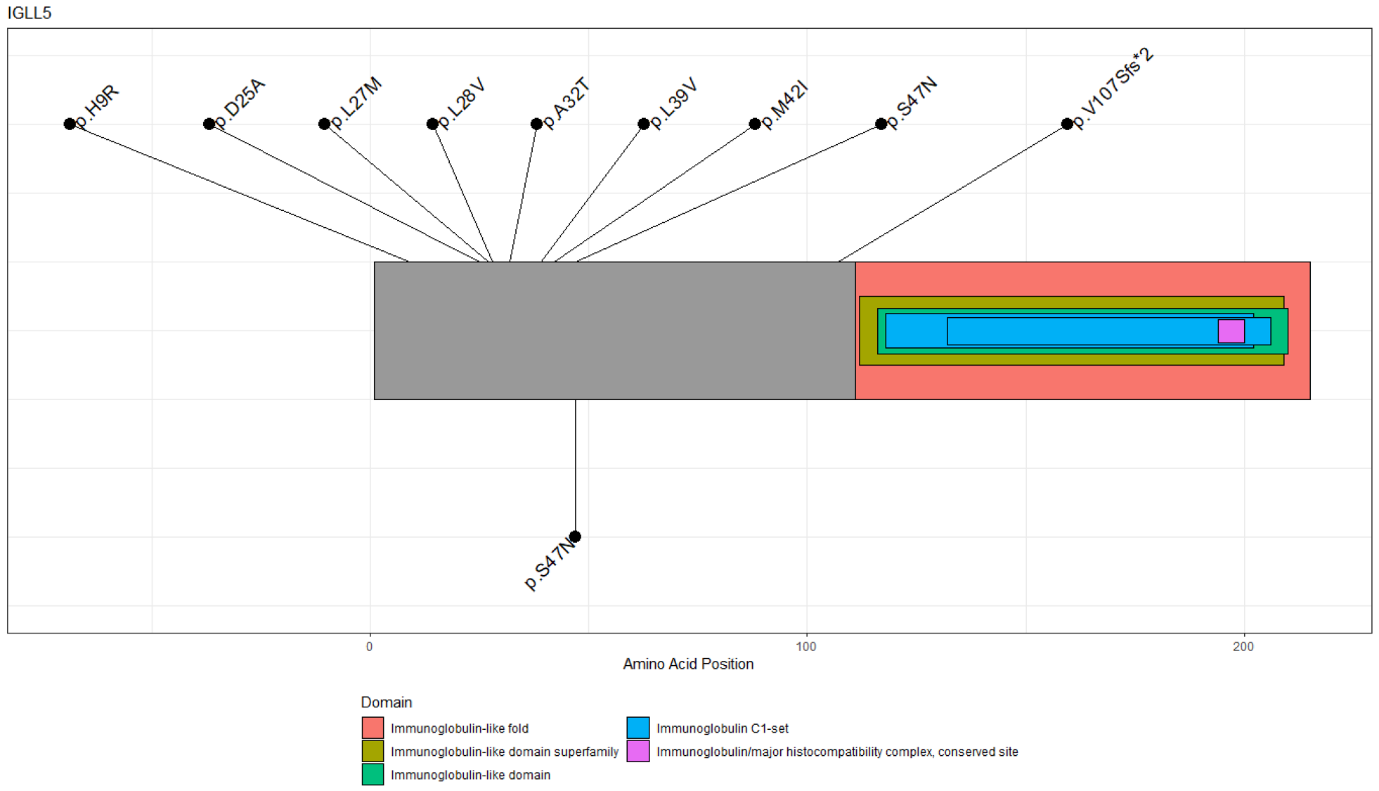
**(E)

(F)

**
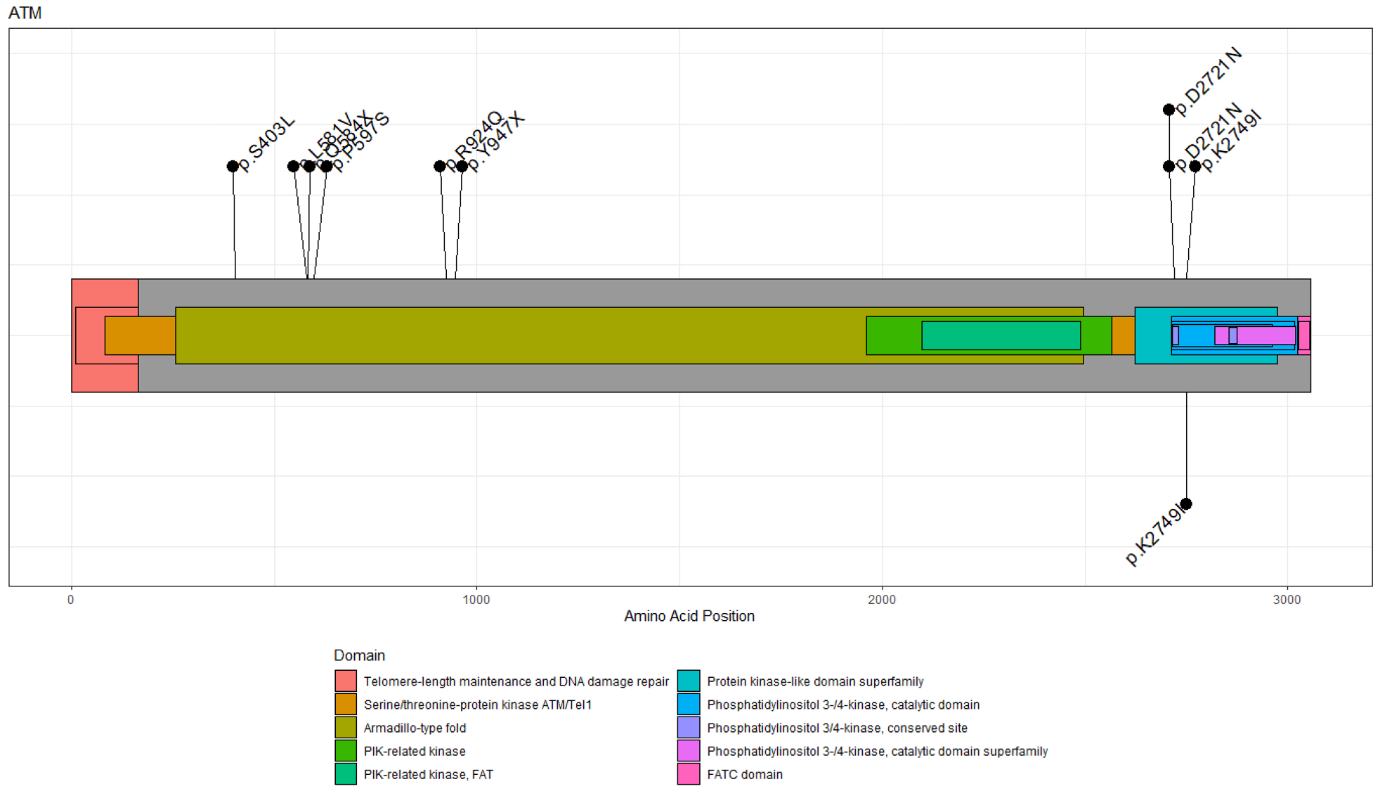
**

(G)

**
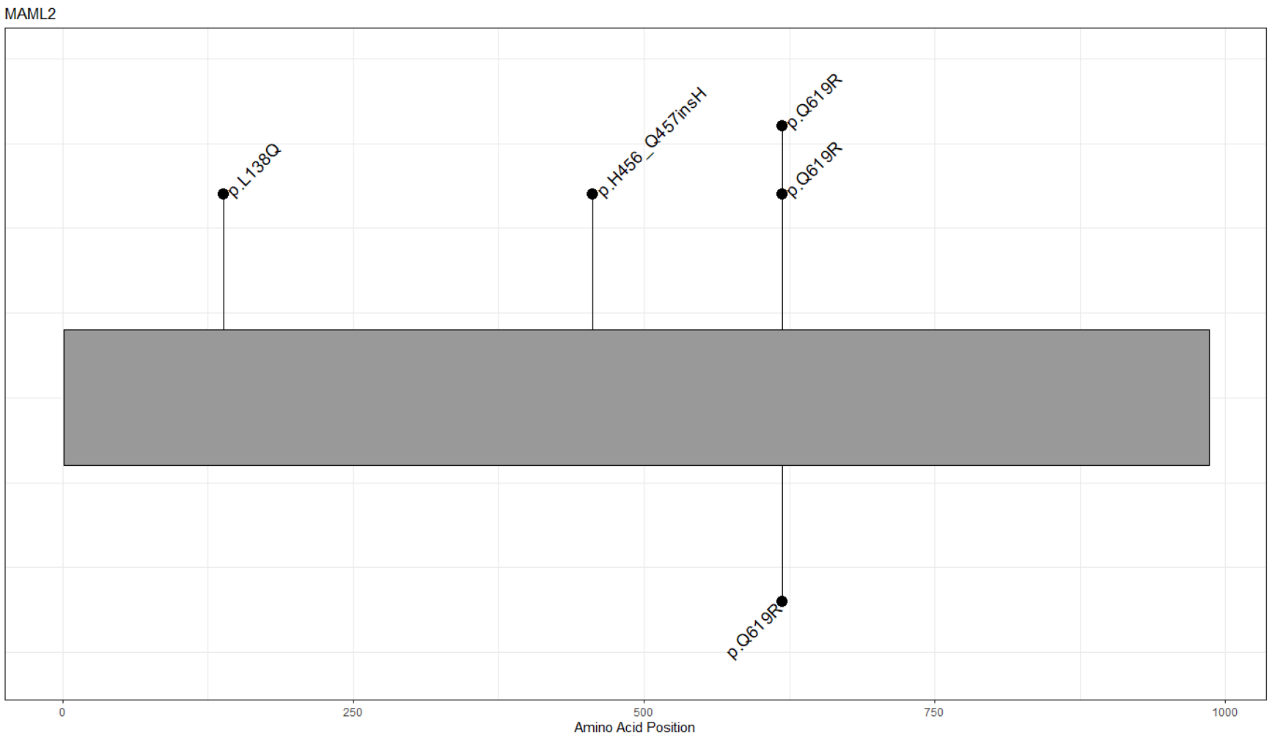
**

**S2 Fig. Diagrams for mutations for most frequent genes in NDMM patients.** (A)-(G) is diagrams for *BRAF, BHLHE41, KRAS, NRAS, IGLL5, ATM,* and *MAML2* respectively. In the case of the *BRAF* gene, p.V600E occurred in 3 patients, and KRAS showed p.Q61R(L) in 3 patients. In the case of NRAS, p.G12D(V) and p.Q61L(K) were confirmed in each of 2 patients. In the ATM, mutations such as p.S403L, p.L581V, and p.Q584X occurred.
